# Combining Wireless Sensor Networks and Semantic Middleware for an Internet of Things-Based Sportsman/Woman Monitoring Application

**DOI:** 10.3390/s130201787

**Published:** 2013-01-31

**Authors:** Jesús Rodríguez-Molina, José-Fernán Martínez, Pedro Castillejo, Lourdes López

**Affiliations:** Centro de Investigación en Tecnologías Software y Sistemas Multimedia para la Sostenibilidad (CITSEM), Edificio La Arboleda, Campus Sur UPM, Ctra Valencia, Km 7, 28031 Madrid, Spain; E-Mails: jf.martinez@upm.es (J.-F.M.); pcastillejo@diatel.upm.es (P.C.); lourdes.lopez@upm.es (L.L.)

**Keywords:** Wireless Sensor Network, semantic middleware, Internet of Things, wearable computing

## Abstract

Wireless Sensor Networks (WSNs) are spearheading the efforts taken to build and deploy systems aiming to accomplish the ultimate objectives of the Internet of Things. Due to the sensors WSNs nodes are provided with, and to their ubiquity and pervasive capabilities, these networks become extremely suitable for many applications that so-called conventional cabled or wireless networks are unable to handle. One of these still underdeveloped applications is monitoring physical parameters on a person. This is an especially interesting application regarding their age or activity, for any detected hazardous parameter can be notified not only to the monitored person as a warning, but also to any third party that may be helpful under critical circumstances, such as relatives or healthcare centers. We propose a system built to monitor a sportsman/woman during a workout session or performing a sport-related indoor activity. Sensors have been deployed by means of several nodes acting as the nodes of a WSN, along with a semantic middleware development used for hardware complexity abstraction purposes. The data extracted from the environment, combined with the information obtained from the user, will compose the basis of the services that can be obtained.

## 1. Introduction

Wireless Sensor Networks play a very significant role in the Internet of Things (IoT); yet in order to clarify the possibilities of WSNs, the concept of the IoT must be examined first. For Huang and Li, it is a network made possible by interconnecting nets related to “things”—deeming “things” as entities people are concerned about—existing around data of products managed intelligently, going as far as claiming that the IoT can be regarded as a specific application form of Semantic Web [1]. Others, such as Coetzee and Eksteen, describe the Internet of Things as a vision where all the objects present in our world can be uniquely identified as part of the Internet, along with their most important information, and can be accessed by the network, impacting dramatically in our professional, personal and social contexts [2]. Regardless of how dissimilar definitions may be, there are several underlying concepts that appear when the objectives of the Internet of Things are defined.

Firstly, the Internet of Things has omnipresence as a leading objective; it aims to have all the elements present in the application context identified, if necessary, by augmenting them with imperceptible electronic machines, providing data about their most prominent current (temperature, positioning, speed) or “historic” (date and origin of the product, assembling processes) features, porting the real element into a ubiquitous dimension where all the electronically augmented objects are present and interact among them. Ideally, networks charged with the task of giving shape to the Internet of Things and the very devices that are part of those networks will be omnipresent [3]. Plus, if the idea of omnipresence is formulated with a synonymous word such as ubiquity, the pivotal role of ubiquitous computing in the Internet of Things can be easily apprehended.

Secondly, an Internet of Things system must fulfill its objectives with *calmness*. On the one hand, it refers to keeping the electronically enhanced environment as similar as it was before the objects were augmented, so the augmenting hardware should not be perceived by human users so as to have them embracing the new entities as the ones human users are used to. If this condition is to be met, used computers and electronics must be shrunk to their minimal expression; otherwise, either they will not be accepted by people as easily, or they will be accepted as a separated, differentiated entity that is not seamlessly integrated in the former object. On the other hand, the newly upgraded object must not demand more attention than the former object did or generate any unforeseen event that the non-upgraded object was not expected to do. If a table, a coat or the tires of a vehicle require an attention they did not require before, it cannot be said about them that they are calm or their electronic parts are fully merged into the object. Seamless integration of ubiquitous components requires that usage and interaction with them is done in a natural and unconscious manner.

Thirdly, the Internet of Things has to offer reliability. Among other features, IoT is expected to be pervasive, that is, to offer information on a daily non-stop basis; consequently, the devices that are deployed under an Internet of Things-based scenario should be working without significant interruptions indefinitely, so in the case there was an issue (a node from a Wireless Sensor Network running out of battery, de-attaching from where it was placed, *etc.*) the deployed elements of the system ought to be smart enough to guarantee the continuity of the functionalities and services demanded by the user of the systems (for example, having another node of a Wireless Sensor Network aware of another node failing for any reason, and assuming the duties the downed node had before) or, in a nutshell, self-healing the system. Many research projects are pointing at the idea of offering reliability to a system or a particular part of it [4].

The Internet of Things must also provide security. For example, if a system is sending and receiving data collected by sensors involving personal and private information, and these confidential data are not send or received under strict security measures, data could get leaked and traded illicitly, consequently making the system not usable for its original purposes. Any implementation under the Internet of Things inspiration must be secured with functionalities that will provide the same degree of security that any other conventional system. This is a topic that has is being tackled by many researchers, either trying to create trustworthy infrastructure to enhance the privacy of the Internet of Things [5] or creating developments secure enough to provide applications on fields as healthcare [6].

In addition to these common underlying concepts on the Internet of Things, ambient intelligence should be considered as well. Due to repeated data retrieval the environmental nodes of an architecture can gain intelligence, meaning by that learning how to best tackle a task and becoming aware to a point of the user intentions. Finally, there is the idea of context awareness. It is what allows a ubiquitous system to acknowledge under which precise and current conditions the system is carrying out its main duties. This is usually achieved by the readings given by its sensors; for example, if there is a system in charge of watering a variety of crops, in case it is able to distinguish between a sunny or a rainy day (and therefore, the luminosity and watering required by the plants) or what crop is the one being monitored (wheat, for example, does not require the same amount of water that corn), then the system is context-aware.

**Figure 1.**
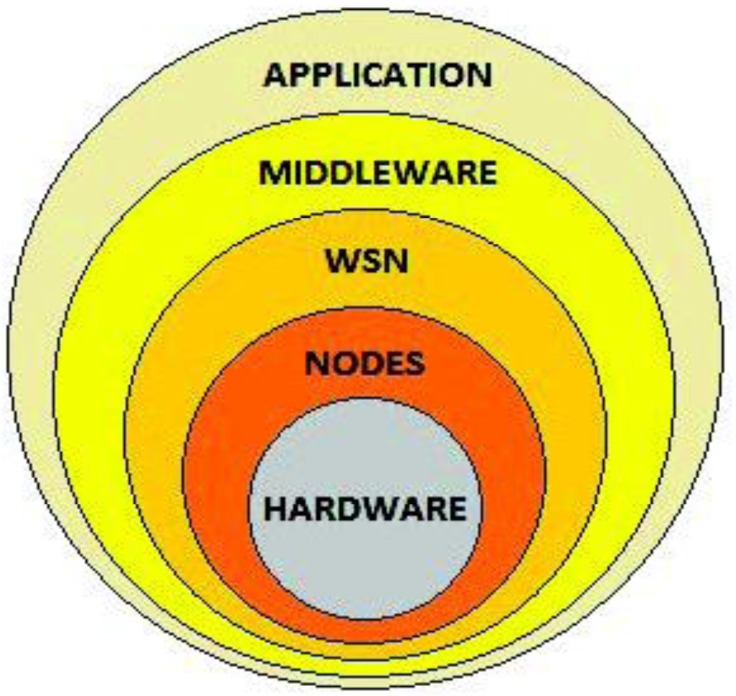
Dependence relations among the main components of a ubiquitous system.

Typically, the different required hardware components in a WSN (sensors, boards, interfaces, radio transmitters, *etc.*) will be merged into a node, and a collection of nodes intercommunicating will compose a Wireless Sensor Network. All the hardware heterogeneity of the WSN will be tackled by a middleware architecture that will finally offer the application developers a homogeneous set of capabilities for them to build applications to be put to a use by a final actor. This ever-encasing process is shown in Figure 1.

Among the many classifications of applications in WSNs suggested by the research community, Yen's, distinguishing among event-driven and random-source applications, is one of the most suitable for our semantic middleware architecture [7]—despite being at the middleware level rather than at the application one since the ubiquitous middleware architecture that is mounted on the WSN in the context of our research project is a random-source application mixing a periodic scenario–where data is requested by an internal node every fixed period of time- and a query- based one where the final human user will request services to the system making use of the Wireless Sensor Network—along with having some elements of non-simultaneous data aggregation routing.

It is only inevitable, though, to perceive that Wireless Sensor Networks, and more generally, the Internet of Things, have not achieved a level of popularity as high as expected. Liu states that “ubiquitous computing's novel nonintrusive human-computer interaction model leads to an intrusive transition process for the majority market including both enterprises and end users” [8]. A general concern about letting loose too much private information if ever interacting with a system of these characteristics does not help either, in spite of the efforts around IoT-based applications pointing at the idea that security and privacy must be guaranteed from the very first steps of a development, rather than as an afterthought or an add-on. Therefore, although services and applications related with the Internet of Things that make use of Wireless Sensor Networks are well-known from a Research and Development perspective, there is a general shortage of them for the end users, who are often not involved in the fields of Information Technology or Computer Science.

This paper presents a model used in a research project named Lifewear [9] (TSI-020400-2010-100) carried out, among many European partners, by the Grupo de Redes y Servicios de próxima generación (Next Generation Networks and Services Group, GRyS) research group belonging to the Universidad Politécnica de Madrid (Technical University of Madrid, UPM) that attempts to fill some of the void present in these applications, aiming to provide services with clear and defined functionalities and to obtain an added value for a ubiquitous middleware architecture deployed onto a Wireless Sensor Network as part of a system based on the Internet of Things.

## 2. Background and Related Work

When referring to the background our project is focused on, there are two topics of main importance: Middleware architectures and their adaptation to a context where data will be collected from ubiquitously placed sensors. The most prominent functionality of the middleware can be defined as “to hide the internal workings and the heterogeneity of the system, providing standard interfaces, abstractions and a set of services, that depends largely on the application” [10]. Besides, as far as Wireless Sensor Networks are concerned, middleware is present as the entity giving support for the development, deployment, maintenance and execution of a WSN application, covering devices and networks connected to the WSN [10]. In ubiquitous computing, middleware has retained its most important functionality–insulation of both the highest layers and the people related with those highest layers (either application consumers or application developers) from the heterogeneity and peculiarities of the lower, more hardware-oriented levels, and by doing so, providing higher levels with an homogenous and abstract environment–taking into account the expectable adaptations that must be faced in order to have a middleware layer performing seamlessly under new conditions and rules. Consequently, if middleware architectures are going to run on machines designed for this ubiquitous context, they must fulfill a series of new requirements not found in more conventional areas:

1. High flexibility and adaptation skills. Unlike regular networks, where number of members, configuration, topology and general features change from time to time at a slow pace, wearable devices and ubiquitous networks are much more dynamic and unpredictable in terms of network topology. A middleware capable of disabling obsolete applications, dropping connections with unreachable nodes or enabling new applications and discovering new pieces of hardware in the ubiquitous network is a must.
2. Reusability for existing pervasive applications. In an area changing its layout and its utilities in an accelerated fashion, many applications (and the means to support them) could rapidly become outdated and, what is worse, unusable. One of the most important functionalities of the middleware layer will be making sure that a generous degree of retro-compatibility is present and not just the latest versions of an application are the only functional ones (it has to be considered that many—if not most of- users will not renew them as soon as an update or an upgrade is available). After all, one of the main reasons for designing early middleware platforms was the correct performance of new applications coexisting with old legacy systems.
3. Interoperability among different platforms. Obviously, if one of the most important missions of middleware is negating the complexity of the physical layer and providing a generic abstraction to software engineers and developers, the differences and boundaries between platforms that may be present will have to be blurred so as to develop new applications which are not limited in their scope by the final hardware device that is below them. Note that since hardware is primarily controlled by the operating system of each electronic device, any interaction with the former will imply requests to the latter, so complexity of the different operating systems must be born in mind.

In order to have a holistic view of middleware architectures, a classification must be offered at this point. There are many different ways to put middleware architectures in order. According to Hwang *et al.* [11], middleware architectures used on Wireless Sensor Networks can be divided into three different kinds: In-network middleware, where the software belonging to the middleware architecture is uploaded into the sensor-carrying devices or nodes, server-side middleware, with the ubiquitous middleware architecture deployed on servers, and hybrid schemes middleware architectures, combining parts of the two former software schemes.

Moreover, Salem and Nader [12] offer another classification where they distinguish between two different main categories: programming support—involved in providing services, systems and runtime mechanisms as safe code execution, reliable code distribution and application-specific services—and programming abstractions—focused on how a Wireless Sensor Network is perceived and how to provide abstractions about sensor nodes and other data. Plus, the authors identify five additional subcategories under the programming support one (related to conceptions of supporting software tools: virtual machine, database, modular or agent-based, application-driven and message oriented middleware) and two more as part of the programming abstraction conception (one bent on describing global behavior and another one dealing with local behavior). The five subcategories belonging to the group of programming support are:

1. Virtual machine: This point of view is composed by Virtual Machines, mobile agents and code interpreters. Compared with the other points of view, they stand out for their flexibility, allowing the programmers and developers to implement applications divided into smaller modules that are able to be distributed throughout the network by the system. The paths of distribution will be taken by previously designed algorithms, bent on reducing energy and resource depletion.
  There are many implementations that can be displayed as examples. For example, Maté architecture was developed as an effort to provide an interpreter that runs on TinyOS as the operating system. According to its designers, “Maté is a bytecode interpreter that runs on TinyOS. It is a single TinyOS component that sits on top of several system components, including sensors, the network stack, and nonvolatile storage (the logger)” [13]. Written in nesC, Maté is able to run the code present on a piece of hardware by making use of two stacks: an operand stack and a return address stack. Typically, Maté will use what their creators call capsules, small chunks of data made of bytecode, identification and version information. This is a good example of how ubiquitous middleware architectures are implemented, but it is compulsory associated with TinyOS, as it is the operating system it uses to run all the higher level applications, thus narrowing its usability.
  Also, a Squawk Virtual Machine can be exemplified as another virtual machine-based solution; it will be executed in Sun SPOT motes from the manufacturer Oracle; not only it can be considered as a middleware architecture but also as an operating system, as it will assume the expectable functionalities of an operating system in these nodes; however, Squawk has been designed keeping in mind Sun SPOT nodes, so its usability is severely restricted for any other device that has no relation with these nodes or Java 2 Micro Edition. Sun SPOT motes are the device chosen as the nodes of the Wireless Sensor Network present in this project, so they will be thoroughly described later.
2. Database: this point of view regards the network as a virtual database. In this conception, a very simple interface is offered for information exchange, using queries to extract information from the sensors placed in the Wireless Sensor Networks. However, it has an important drawback: this point of view does not offer any support for real-time applications, providing only estimated data, making impossible the inferring of relations between space and time between events.
  An implementation of this kind of programming support is Cougar, for the whole Wireless Sensor Network is just a relational database under this programming model, which works by means of user queries, where an optimizer is charged with the task of generating an efficient query plan for in-network query processing [14]. Operation management of the Wireless Sensor Network is carried out with a query language similar to SQL. Cougar is able to characterize any node of the network as a database generating structured registries with several fields. Abstract data types are used to model signal processing functions, and an energy saving system based on request distribution among the nodes will be employed as well. Another case is TinyDB, developed by Madden *et al.*, and defined as “a distributed query processor that runs on each of the nodes in a sensor network” [15], meaning that TinyDB makes use of a queries processing system in order to get the data from the Wireless Sensor Network while running the code on each of the nodes. It is not a coincidence that it shares part of its name with TinyOS, as this is the operating system TinyDB is mounted upon. TinyDB keeps a virtual database with information about the kind of sensors that are being used, node identification and the battery charge that still remains for each of them. Furthermore, it will employ an interface similar to SQL's to better extract the required data. However, TinyDB is limited by its mandatory use of TinyOS; no other operating systems are allowed, and the code uploaded to the nodes must be written in C as the programming language.
3. Modular programming (agents). This approach uses the mobile nature of the code under its principles to its advantage. As it happened with the Virtual Machine approach, the modularity of the applications makes it easier to inject and distribute mobile code, and propagating small modules throughout the network makes the applications less energy-demanding.
  As examples of this programming model, Impala and the Smart Messages Project can be put forward. Impala was first described by Liu *et al.* as an architectonic model that offered mobility, transparency and quality changing from one protocol to another and adapting applications in real-time, thus preventing many node failures and runtime errors [16]. Smart Messages Project, on the other hand, is based on code and data contained by message-like agents that is capable of migrating throughout the Wireless Sensor Network [17]. Smart Messages Project puts forward a distributed model called Cooperative Computing, where migratory execution units called smart messages are defined to cooperate for a common goal.
4. Application driven. Here, unlike the other points of view, an architecture following a stack model is given. This is advantageous for developers, as it offers them the chance to tune up the application to a level unthinkable with other solutions. Since the network management is done by the applications themselves, Quality of service or QoS can be more easily managed and improved, according to the needs of the application.
  An application driven programming model is MiLAN (Middleware Linking Applications and Networks). MiLAN focuses on high-level details, using a characterized interface, and allowing network applications to specify their particular needs of Quality of Service (QoS) and trim network features in order to optimize its performance without ignoring the needs of QoS [18]. Commonly, MiLAN will select the group of nodes that are compliant to the specifications of the QoS required by the application running at that moment by using network plug-ins that will determine the group of nodes that better accomplish the requested duties. Under MiLAN, networks can be configured in a very accurate way, due to the fact that the group of nodes is chosen by making use of its extended architecture (comprising the network protocol stack, the abstraction layer and the network plug-ins) and specialized graphics responsible for adding changes based on the needs of the applications. MiLAN is one of the most application layer-focused presented middleware architectures.
5. Message-Oriented Middleware (MOM). This is the model that probably best suits programming under Wireless Sensor Networks conditions. A mechanism of publication and subscription to services (publish-subscribe model) is put into practice in order to facilitate communications between nodes and base stations. Plus, this model enables asynchronous communication, allowing for a flexible model of communications between information creator and receiver.

Two representative implementations of this kind of model are Mires and SensorBus. Mires makes use of activation messages to put to a use a communication infrastructure based on a component used for publication and subscription (or more accurately, a publish subscriber service). This component will synchronize communications among middleware services and will make the system work properly. In addition to this, a routing component and a data aggregation service are offered; this latter service lets the user point out both how the data is going to be added and the relation between harvested and added data [19]. As it happened in TinyDB case, Mires makes use of TinyOS as the operating system, although it uses C as the programming language. SensorBus, on the other hand, implements the publication-subscription paradigm in an anonymous, asynchronous and multicast way [20]. SensorBus makes use of a key component called events producer that will publish the kind of events that are going to be available for the event-consuming components of the system. The event-consuming component will just subscribe to it and will receive notifications from the event generators about the kind of subjects taking place. On the other hand, other categories falling under the orbit of programming abstraction are:

1. Global behavior or macroprogramming. This point of view tries to consider the global behavior of the whole Wireless Sensor Network. Instead of programming having only individual nodes in mind, the Wireless Sensor Network is programmed as whole, black-boxed subsystem, with its expected behavior as a whole according to a high-level specification that generates automatically the code that enables the behavior of every node, thus freeing application development from treating the low architectural levels of the nodes that compose a network.
  Two examples can be put forward belonging to the macroprogramming perspective: Kairos and Semantic Streams. Kairos makes use of the concept of “divide and conquer”: the global behavior of the whole network will be broken into entities called subprograms, and these latter can be executed locally in every node. Since this process will be carried out not only in compilation time, but also in running time, the developer is left charged just with handling a few primitives. Kairos attempts to describe the global behavior of a Wireless Sensor Network under a centralized perspective, as opposed to other middleware architectures that, according to its creators, focus only on how the behavior of one local node [21]. In addition to this, Kairos makes possible the selection of the way the processes of each node are synchronized: either in a more flexible manner (loose synchronization) or in a strictly defined one (tight synchronization). It is up to the programmer to decide how to use these processes to have an efficient network without any system overload.
  Semantic Streams, on the other hand, proves to have more intelligence than the other programming perspectives: it enables the user to make declarative requests about semantic interpretations of the information gathered by the sensors, where data has a meaning inside a context. However, it has to be taken into account that although it makes an extensive use of the capabilities of sensors present in an environment, Semantic Streams has been conceived to be used with mostly non-moving sensors, either wired or a hop away from the base station [22].
2. Local behavior (or geometric, data-centric). This point of view takes the opposite path: nodes are taken into account individually, inside the distributed network. This local behavior is focused on two features: firstly, the nature of the data info obtained by the sensors; secondly, the specific location of the network element. Any request for a data reading (moisture, temperature, luminosity) of a particular spot inside the environment could be an example of this category.

Another two examples following local behavior programming paradigm can be offered here: Abstract Regions and EnviroTrack. Abstract Regions was conceived by Welsh and other researchers in 2004; it is nothing more than a group of communication, general-purpose service primitives that provides data aggregation, addressing, data sharing and reduction in the local regions of the network [23]. Other of the main ideas behind Abstract Regions is shifting the focus on local computation to the detriment of radio communication, thus reducing the bandwidth used in communications (at the expense of requiring more processing activity). Data processing and aggregation are done at the local level by a group of cooperative nodes that communicate among them. As far as a hierarchy is concerned, data are sent from the nodes to the base station without any go-between, in another effort to reduce bandwidth depletion. Any object that requires a very precise tracking, which will aggregate readings from all the nodes nearby, is an example of how a system could benefit from this implementation. EnviroTrack, on the other hand, was designed by Abdelzaher *et al.* in 2004. It tries to create a data-centric inspired naming system called “attribute-based naming” that makes use of context labels [24]. This paradigm is original in the fact that addressing and routing are not based on the destination node, but on the content of the requested data. The dynamic behavior of a mobile object and the nodes that belong to the network as well can be accurately followed, making this system fairly suitable for tracking or environmental monitoring solutions. However, as it happened in other examples of programming paradigms, EnviroTrack makes use of TinyOS as the operating system, and therefore it is limited in the usage of programs.

In addition to the middleware architectures fitting under the classification of Hadim Salem and Mohamed Nader, there are three other remarkable legacy solutions that are interesting to consider and do not fit properly into the already exposed description. Aura, to begin with, was developed in 2002, and had as its main objective to keep services available for the user no matter where they were placed at that very moment, even and especially if that means reconfiguring dynamically a task already interacting with a human user. The first glimpses of this architecture appeared while conducting research activities driven to create some sort of middleware architecture capable of a successful performance in a ubiquitous computing environment [25]. Having chosen Aura as the name of choice is not devoid of meaning: this middleware architecture will create an area around the user (the Aura) wherein information and computing services will become persistent, in spite of their location, and therefore executable at any place. Obviously, as there are many different areas in a ubiquitous environment, with electronic devices of potentially very different nature and performance, if the current and running services are desired to be kept accessible, the Aura will have to adapt to the available resources of the environment, thus moving around the representation of the task, thus adding a self-tuning feature to this middleware architecture. What is more, Aura also aims to anticipate to the actions of the user, enabling service prevision to a degree.

Secondly, Gaia [26] shares many of the concepts conceived by Aura architecture, especially the ones related to the challenge of adapting data and applications to the possibilities of the current devices that are providing them. For example, where Aura made use of an Aura to name the place in space where all the services would be stored and interfaced, Gaia is implementing a “Smart Space” where once it is active—turning into what is called an Active Space, the applications and services will be dynamically mapped according to the available resources of the device that uses them, as it was done when using Aura. An Active Space is composed of a cluster of devices that have a strong relation with the final user and all their software capabilities and resources available, from operating systems to applications. Besides, Gaia was also capable of providing functionalities used to customize the available applications, an allowing users to move through different active spaces as well [27].

Thirdly, the more popular Universal Plug and Play (UPnP) is an architecture first issued in 2006 initially conceived as an extension of the plug and play concept into a networked, wireless environment, it ensures openness and independence to a degree: UPnP is an open source, open architecture which does not depend on platforms or languages for its right performance, while following and making use of some of the best known standards of industry, such as HTTP, SOAP and XML. The target context for UPnP is mostly either a home or a SME environment, where all the devices belonging to the building they are present are interconnected by means of this architecture. UPnP intends to integrate all the devices found in these scenarios seamlessly, without making any intervention from the user necessary (if so, in a plug-and-play interaction fashion). Thus, UPnP can be used to become part of a more complex system, thus interacting with other technologies such as ZigBee [28]. Currently, there is a development called Universal Plug and Play-User Profile (UPnP-UP), aiming to provide user authentication and authorization in pervasive systems security mechanisms absent in the former UPnP [29].

There are some other developments involving Service Oriented Architecture or SOA that are noteworthy, for they show different perspectives of how to measure vital parameters by using semantic middleware architectures. Coronato introduced Uranus, an architecture aiming to be a general oriented service middleware capable of providing ambient assisted living and vital sign monitoring applications [30]. Uranus has been conceived to be used either in an indoor or an outdoor scenario; hardware resources used in Uranus involve PDAs, RFID tags, oximeters, ECG sensors and Zigbee enabled—and, by proxy, 802.15.4 enabled- devices that will be used either indoors or outdoors, depending on where the patient supposed to wear them is present. In order to establish communications, radio transmission technologies guaranteeing a permanent monitoring of the patient will vary from Wi-Fi (indoors) or GPRS (outdoors). Two use cases long term patient monitoring and smart hospital built with radioactive rooms show well how Uranus is capable of being used in heterogeneous scenarios; however, when compared to our system it does not fit that well considering our own use cases: PDAs cannot be regarded as low capability devices, and their clumsiness and power consumption may be higher than desired if they were used by a sportsman/woman. Plus, no services in Uranus middleware architecture are based on ontologies, while our system does, so any effort to interconnect our semantic middleware architecture with others may be easier and more semantic-oriented. Lastly, despite the general orientation—and probably due to it of Uranus middleware, having to program a small, albeit significant, percentage of code lines to have a full adaptation for each of the use cases does not look like a complete seamless adaptation; no reprogramming has to be done in our system once the nodes are deployed, although it is supposed to be used under more specific placements.

Fortino *et al.* put forward their *SPINE2* (an evolution of a previous Signal Processing In-Node Environment SPINE) architecture as a way to develop platform-independent collaborative Wireless Body Sensor Networks [31]. Creators of SPINE2 focus on the idea that there are still very few methods to create platform-independent applications for Wireless Body Sensor Networks, and with their system they are able to create an independent environment for C-like platforms. SPINE2 creators refer to the duties that have to be fulfilled as tasks, with the essential functionalities of Wireless Sensor Networks implemented in each of the tasks (SensingTask for sensing operations, TimingTask to define timers over tasks and FunctionalTask for functional purposes: processing, aggregation and transmission). This approach comes in handy when implementing a Body Area Network, but it does not take into account the interaction of the BAN with other elements, or whether the BAN is encased as part of a broader system that is using a Wireless Sensor Network. Besides, in our system we are using nodes programmed in J2ME, and therefore this environment cannot be used with the hardware devices that we have as nodes at the Wireless sensor Networks.

Corchado *et al.*, on the other hand, suggest a platform called Services laYers over Light PHysical devices (SYLPH) as a healthcare monitoring system [32]. SYLPH implements a stack of layers with two main components to be highlighted, that is, SYLPH Services—used for intercommunication between the interfaces of the nodes belonging to the Wireless Sensor Network and SYLPH Directory Nodes—used for service discovery. As it is done in our system, SYLPH makes use of gateways to interconnect Bluetooth and 802.15.4 related technology (in this particular case, the higher level implementation or Zigbee), and there are many devices resembling our deployment, like several 802.15.4 related devices being part of a Wireless Sensor Network, and Bluetooth sensors worn by a human being. However, this proposal seems to be used for monitoring purposes, along with alerts extracted from monitoring activities, so active service requests from a human operator are not possible under this platform, nor it seems it was an objective for the developers of SYLPH.

Junilla *et al.* try to offer a system with a holistic view that will include as many potential users as possible; they are making use of a platform called UUTE that was used as part of their own research project [33]. When the monitoring platform was developed, there were several choices taken that somewhat mirror ours: Zigbee communications were chosen exclusively instead of a Bluetooth/Zigbee combination as the wireless network technology (in this project, Bluetooth was deemed as a standard consuming too much energy) and ChipCon CC2420 as the radio interface for the Wireless Sensor Network. A common sensor interface was created, along with a client and a server in order to have a functional client-server architecture. Besides, a plethora of sensors, either purchased or tailored, were used: an intelligent pedometer, a hear rate sensor, a blood pressure monitor, a bed sensor, *etc.* Two different use cases were employed for the subsequent testing: A 70 year old woman living alone in a two room apartment and a hip surgery rehabilitation patient, who would have several vital signs measured (blood pressure, weight, *etc.*). All this thorough and detailed work, though, was not conceived to be used in use cases such as ours—a sportsman/woman in a gymnasium-, although the idea of monitoring a person is especially linked to what we do in our own system.

Another platform resembling what has been done in our research project is LOBIN, based on e-Textile and Wireless Sensor Networks for healthcare monitoring [34]. Its creators emphasize the idea of using Wireless Sensor Networks as a mean of having non-invasive procedures of providing healthcare. It is made up by four different domains: a healthcare monitoring subsystem–a set of smart shirts able to collect and process physiological parameters, a location subsystem–made of a beacon point infrastructure, a Wireless Sensor Network subsystem—transmitting data *ad hoc* by using a gateway and a management subsystem—an infrastructure that will be handling the information of each patient. Tests made were involving the subsystems present in this proposal (healthcare monitoring, location and Wireless Sensor Network) and overall so as to check the performance of the system, proving to provide reliable results. LOBIN platform is an accurate solution to have measurements from one patient, although it somewhat neglects the contextual information about the environment, for the beacons and distribution points (BPs and DPs in the paper) are only routing information from one domain to another, rather than using sensors to collect data. Plus, the *ad hoc* methodology that is used is less capable than the hierarchy developed in our system.

Perhaps one of the most holistic views of a system like the one we are going to deal with is the one suggested by Triantafyllidis and Chouvarda, claimed to have monitoring, status login and social sharing functionalities [35]. Apart from the expectable sensing devices and a Service Oriented Architecture or SOA, micro-blogging services have been added to this system too. Therefore, there are three main functionalities that are taken into account: patient monitoring–by using event-driven patterns corresponding to a threshold configuration-, status login–where the information transmission is initialized by the patient by using status descriptors as problems/symptoms, activity or time and location and social sharing—with patients sharing details about their personal health information through their networked community. The architecture of the system has been structured as requiring four different kinds of nodes: A mobile device working as a Mobile Base Unit or MBU, sensors, the backend platform and the external social network platform. When the system was implemented, hardware devices were used: a mobile phone for the MBU and backend subsystems, Twitter as the microbbloging service and a belt capable of taking physiological measurements that, incidentally, is an earlier version of the one that has been used in our deployment. In one of the figures of this article, 50 and 120 appear as lower and upper heart rate values, so they have been used as thresholds for heart rate in our own system. Despite being an ambitious platform and offering novelties when compared to other ones, we believe that there may be some issues regarding information privacy if vital sign parameters are published in a microblogging network.The last proposal that we would like to mention is the one from Abousharkh and Mouftah, focused on creating a middleware for Wireless Body Area Networks that will improve their usability [36]. Not in an unusual way, this middleware architecture will be made of three subsystems: a medical sensor network gathering information from any person or patient, a wearable device such as a smartphone as a central coordinator node and a central server used to store sensor signals or send the patient information. Eight different kinds of messages have been defined in this middleware architecture The idea of using a gateway to coordinate a Wireless Sensor Network, Web services to establish communications, and an overall SOA-inspired architecture resemble our own proposal, albeit ours goes beyond a WBAN, as it takes into account environmental temperature from different points of the place it is deployed into.

## 3. Description of the Scenario and the Available Services

The semantic middleware architecture used for the system that is going to be shown has been developed taking into account the special needs and constrains when coexisting with a ubiquitous, pervasive environment:

- It has been designed with the idea of using it on low-capability devices: typically, it will be uploaded on nodes participating in a Wireless Sensor Network, so any device with node-like or higher capabilities with interconnection functionalities will be able to execute it with very little trouble.
- It is a software-centric middleware: the kind of devices on the physical layer, except for its obvious middleware objectives related with hardware abstraction, is not considered by the application layer. Instead, it is composed by a series of modules with delimitated tasks within its architecture, as it will be seen later. Additionally, its inner architecture makes use of software agents that will manage sensor-equipped hardware devices. Each agent has different duties that, although usually dissimilar, can be federated to have a cooperation grid among several of the agents, seeking the successful delivery of a composed service. These agents are closely linked to what the place they are present can offer: either data collected from sensors: temperature, luminosity, *etc.* or the possibility to trigger an actuator like a loudspeaker, LEDs, *etc.* The agents are responsible for considering the environmental information as a major element present on Wireless Sensor Networks in order to make the system more context-aware, as other developments pointing at that direction also do, such as FamiWare [37]. The concept of having a software-centric point of view stress the importance of software contents rather than any particular piece of hardware, unlike other semantic middleware architectures. For example, Picone *et al.* put forward a mobile device-centric architecture around mobile devices that collect data from sensors [38], but in our own semantic middleware architecture mobile devices are used as RESTful elements that are able to request services wirelessly.
- It is part of a bigger framework based on Service-Oriented Architecture and Service-Oriented Computing principles. The main goal of this semantic middleware architecture is providing services to a final user; to accomplish this, it will be part of a layer that can be regarded as the Service-Oriented Software Platform, placed in a model for service with layers above and below.

### 3.1. Service Ontology Description

An ontology is a formal and semantic representation of a set of concepts along with the relationships between those concepts within a domain. An example of a semantic solution designed for ubiquitous applications is Standard Ontology for Ubiquitous and Pervasive Applications (SOUPA) [39]. SOUPA is a proposal that defines core concepts by adopting several consensus ontologies. Some concepts defined in SOUPA ontology were used to model the context information within the Lifewear project.

Some of the concepts re-used (so as to describe the environment were the user is located) are the OpenCyc Spatial and RCC ontologies, which include SpatialThing, that is related to LocationCoordinates class. These ontologies have been extended with the Location class to describe the different areas that compose an environment by using a symbolic representation with more information for the user (*i.e.*, muscle room, aerobic area, *etc.*). Moreover, the term EnvironmentProperty has been included to describe the properties (e.g., lighting intensity, presence detection, temperature, *etc.*) of a certain location.

Also, the terms Service, ServiceCategory, Operation, Argument, and Process were defined in order to describe the system. The central term is Service, which represents the services (e.g., Alarms, Heart Rate, Body Temperature, *etc.*) that the system provides. There have been defined several categories to classify the Services, which are described with the following information: Profile (the public description of the service), Process (the logic of the service) and Context (the context conditions in which the service is provided).

**Figure 2.**
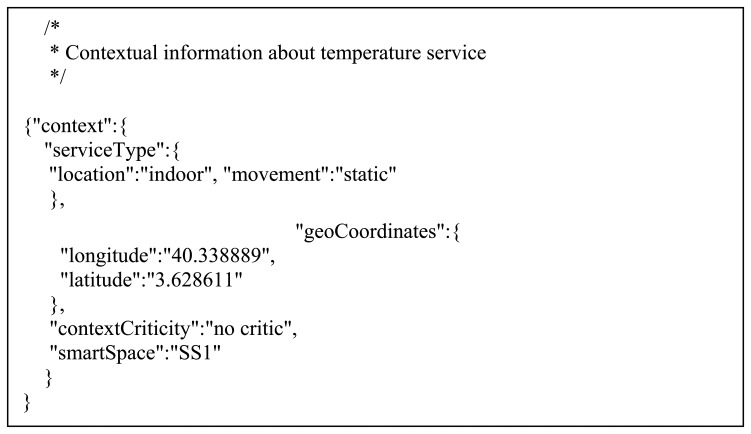
Ontology example.

The term Person, defined in the FOAF SOUPA ontology, has also been used in order to describe the users of the system. This term is described by a set of properties that include profile information (e.g., name, gender, birth date, *etc.*), contact information (e.g., email, mailing address, phone numbers, *etc.*), and social and professional relationships. In order to upgrade the description of the Lifewear users, the class UserProperty has been added. This class represents some user's properties, such as user preferences (e.g., exercise routines, personal marks, thresholds, *etc.*). Some applications need to track the user's position in the exercising area. To fit these requisites, the currentLocation relationship was defined. Finally, a person is also associated to policies. A policy represents a set of operations and services that a user is allowed to use. The policy also describes the context information that a person can see and/or modify. It is remarkable that the context concept defined here is related to the services, and not to the user. A service context includes the location of the service (indoors or outdoors), the accuracy of the sensor, the units of the measure, *etc.* The described ontology was implemented using the Web Ontology Language (OWL), a W3C standard ontology markup language that facilitates knowledge automated reasoning. An example of this ontology is displayed in Figure 2.

### 3.2. Working Model Overview

**Figure 3.**
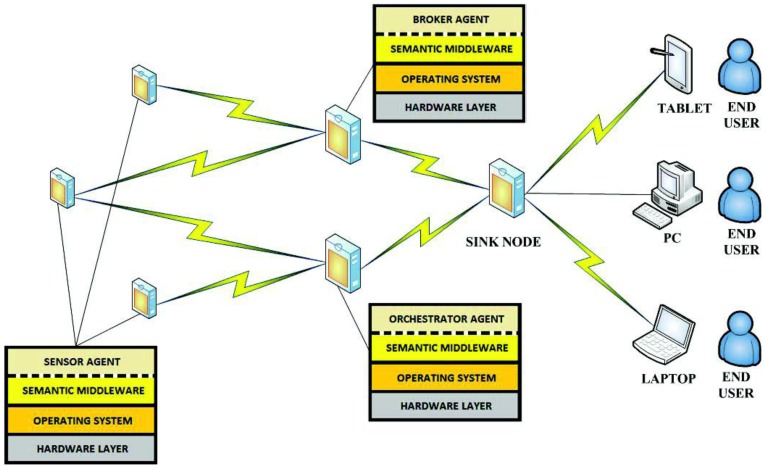
Internetworking model within the Wireless Sensor Network.

The semantic middleware architecture presented here does not work according to a flat model where all the nodes are communicating as if they were an *ad-hoc* grid but has an inner hierarchy; its main components are shown in Figure 3. It is of critical importance to understand that the nodes presented here (wearable sensors and personal devices, Broker, Orchestrator and Sink) are playing those roles because there are software agents deployed on them (Orchestrator agent, Broker agent, *etc.*) that fulfil the tasks expected from their kind. What determines the functionalities of nodes in a Wireless Sensor Network is not the node, but the software uploaded on the node regardless of other considerations (for example, it is possible to have several agents uploaded on a node, but if one of the agents is the Orchestrator agent, the node will assume the role and the responsibilities of an Orchestrator node, apart from whatever tasks are associated to the other deployed agents on the node).

By having software agents that will only be changed when a different one is uploaded, the nodes are assuming fixed roles that cannot be modified; nevertheless, several agents—each one with a different role- may be uploaded a single time, so several roles could be performed simultaneously at the same node, since the role is performed by a software agent uploaded to the node, rather than the node itself, and a node can have several different agents. This is the case of the node that has the Orchestrator agent; although it is the most prominent agent of the node—hence, having the node assuming that role—the same physical node has uploaded one more agent, the Orchestrator Alarms one, so the roles of service orchestration and alarm triggering at the first stage of alarm communication, are performed at the same node by different software agents. Should it be useful for the system, a single node could have Broker and Orchestrator agents.

#### 3.2.1. Base Station/Gateway/Sink

The base station plays a role similar to a gateway in other kinds of networks: it will be the gateway between the Wireless Sensor Network and the conventional network. Under a more physical point of view, the Gateway (also named as Base station and Sink) will be sharing features from the two worlds: it will usually be a ubiquitous device capable to interact with a non-ubiquitous device in charge of processing the data retrieved from the Wireless Sensor Network; as an example, the device used in the scenario created for the Lifewear research project is a Sun SPOT base station, a special node that, while able to communicate with all the other deployed nodes, it also makes use of the code that is located in a PC, and it is attending requests coming from an ESB deployed at the same Personal Computer. In this case, the base station is plugged to the PC via a USB interface, and since it has no power on its own, must remain connected all the time to attend requests.

This is a unique case in the semantic middleware architecture in the sense that there is not a particular agent deployed at one node, and the hardware is playing a more prominent role than in all the other elements from the Wireless Sensor Network. However, judging from the fact that this element has no other functionality that working as a gateway (from a paradigmatic point of view, carrying data from one environment to another), sink (from a data perspective, since it is going to receive all the information from the WSN) or base station (from a system point of view, as one of the elements involved in it) it is not necessary to upload an agent on it, since the required data conversions are done by using hardware rather than software.

Unlike the other software agents, a sink is an element that will almost always be participating of a Wireless Sensor Network, regardless of its inner architecture. Although there are some deployments that use multiple static sinks to connect legacy networks [40] or mobile sinks that may require of control mechanisms [41], in the scenario used for the Lifewear research project one single sink is enough for its satisfactory performance.

#### 3.2.2. Broker Agent

The broker agent is a recurring part of middleware architectures on Wireless Sensor Networks. It is put forward as a major agent in several developments; for example, a model of virtual brokers for large-scale operations regarding publication and subscription to services in WSNs has been put forward [42], and our own models use sensor virtualization as a core concept. In our WSN architecture, the Broker agent is probably the most important element of the WSN once it is loaded with semantic middleware architecture. This is due to two reasons:

- Any new service that springs up in the Wireless Sensor Network will send a notification message that will be received by the Broker, so it keeps all the available services registered. If any request for a service is done by the user, it will have the Broker as its first (or second, if the base station is taken into account) step in the WSN, as the Broker agent redirects the request towards the node that is capable of providing the service. It also happens the other way around: when the answer is leaving the WSN, the Broker will direct it back to the base station as the last (or second to last) stage of the communication. If a new node with new agents capable of providing new services appears while the other members of the Wireless Sensor Network are operational, the agents with their services will be registered as any other that had been formerly done before and the services become fully available and reachable. If for any reason a node becomes incapacitated (battery depletion, physical crashes, *etc.*), when human operators request the service they will be told that it is unavailable instead of having it replaced, for simple and composed services are dependent, among other criteria, on environmental temperature values, and they may differ greatly from one node location to another.
- When a composed service is requested, as it will be seen in the next part, the Broker agent will manage all the inner interchange of messages that may be needed, not requiring any further work from the human user.

Therefore, according to its functionalities it can be imagined that if the Broker agent becomes incapacitated, the whole middleware architecture will be unable to work normally, so the Broker agent must always be uploaded to the node in best conditions (best radio signal, highest battery level, *etc.*).

#### 3.2.3. Orchestrator Agent

Orchestrator agent is critical to correctly provide composed services; without it, they simply cannot be delivered. Usually, the Orchestrator Agent assumes the role of publishing the composed services present in the Wireless Sensor Network. So, when there is a request of this sort of service, the Broker agent will re-route it to the Orchestrator agent. In fact, though, the Orchestrator does not collect any data required for a composed service, but it is aware of the simple services that are needed, so it will ask for them to the Broker agent until it has all the required data, calculates the value from them, and sends it back to the Broker. To a lesser extent, Orchestrator agents appear as part of middleware architectures in some middleware architectures, or are taken into account either when external instructions or node-binding operations are involved in Wireless Sensor Network communications [43].

#### 3.2.4. Sensor Agents

These are the agents located on the nodes of the Wireless Sensor Networks that are devoted to information harvesting. Although these agents are still pieces of software, the services they represent are dependent on the data that is collected from the environment; therefore, agents are closely linked to the sensors that the device they are uploaded on has mounted (unlike the Broker or Orchestrator agent, that perform purely software functionalities regarding data request and delivery).

Sensors are the component of the Wireless Sensor Network that is most closely in touch with the outside world. Commonly, context data from this outside world is retrieved by means of the sensors measuring the data, and the software sensor agents managing the sensors will be porting the physical data to a logical format that can be transferred through the system. These agents can be expected to work as mobile agents in a hierarchy [44], performing functionalities that may even come close to the results obtained by more conventional environments, at least under simulated conditions [45].

All in all, they will provide readings from data belonging to the environment they have been placed. In our scenario, information regarding indoor temperature was collected (although depending on the used node, luminosity, humidity and other information can be retrieved too, for they are tasks depending on the existing sensors), as well as data provided by an agent used to port Bluetooth-formatted information from an electronic belt, as it will be exposed later.

### 3.3. Services Offered by the Semantic Middleware Architecture

Once a general glimpse of which the main components of the semantic middleware architecture are, a description of those services will be offered, and right after it, all the services available in the system.

#### 3.3.1. Simple Services

A simple service is a service that is offered by an existing agent, usually uploaded in a Wireless Sensor Network node. It is related with a piece of information provided by a sensor, and will just deliver it when the agent is requested to do so. The information provided is not stored in any intermediate component, but it will be given to the agent that requested it in the first place; a simple service will be a process where the sensor will tackle a reading at the environment and will take the result to the operating system (or in our case, the mini Java Virtual Machine Squawk) which is the entity that will send it to the middleware as the answer that is expecting the application layer. These are the most usual services that will be obtained from a Wireless Sensor Network; depending on the nodes or the semantic degree of the middleware architecture present in the Wireless Sensor Network, some others may be retrieved, such as localization, coverage or even information storage [46].

#### 3.3.2. Composed Services and Sensor Virtualization

A unique feature of this semantic middleware architecture is that it is able to offer one single service based on different readings collected by the Wireless Sensor Network. In this case, the service will not be provided as a result of a request to an agent that, in the end, is consulting a sensor, but as the result of a data processing action, where the data to be processed has been collected by doing several inner requests for different simple data—that is, collected from the sensors associated with the agents, without user intervention. These requests are necessary because the response that is going to be delivered is dependent on the values that have been gathered as the data from the simple inner requests, and the service that is attended this way is a composed service. For the final user, the service is provided as if it was a simple service with no difference with the others. Furthermore, when the Broker agent directs the request for this service to the Orchestrator agent, it is done like this because the Orchestrator agent has published the composed services as if they were simple services that would be attended by “listening” a sensor reading.

#### 3.3.3. Simple Services Communication Model

**Figure 4.**
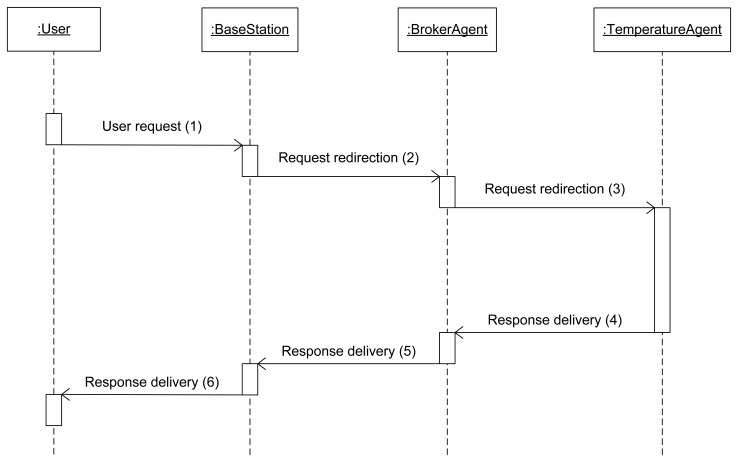
Sequence diagram for a simple service.

The sequence diagram that describes a simple service communication is portrayed in Figure 4. In the sequence diagram a temperature request (which can be invoked in our scenario) is used as an example for how simple services work under our semantic middleware architecture: when the human user requests a temperature, the request is introduced by using a keyboard, a touch screen or whatever device is available at the moment and is redirected via a conventional network (wireless or not, depending on the device from it was made) to the base station (1). After that, as long as the requested service is available in the system, the base station will always redirect it to the Broker agent, the first step on the communication (2), as it is the entity of the WSN that is aware of what services are functional. If the service is recognized by the Broker agent (that will be uploaded on a node of the WSN, most likely a node), the request will be redirected again towards the node that has installed the agent (in this particular case, the temperature agent) capable of taking the data from the sensor present at the piece of hardware (3).

Once the information has been obtained, it has to be delivered to the human user. To accomplish this, the service response will undo the route done before by the request. To begin with, the answer will be carried to the Broker agent, since all the communications are done by following a hierarchy where the Broker will receive all the responses to the requests (4). Once the Broker has received the value, it will be transmitted back to the base station (5), and the base station, bridging the Wireless Sensor Network and the non-ubiquitous network, will finally send it to the device where the final user started the request in the first place (6). The sequence shown here is located at the application layer; the vehicle to transport the requests and answers are JSON messages. JSON is more useful than XML under this ubiquitous environment because, although XML supports standardizing schemas, it is usually more verbose than JSON, consuming more bandwidth and energy resources.

#### 3.3.4. Composed Services Communication Model

**Figure 5.**
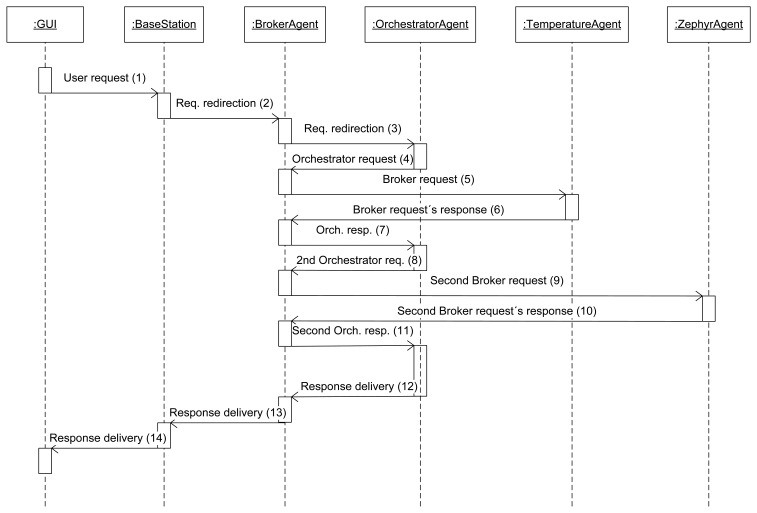
Sequence diagram for a composed service.

The sequence diagram that describes a composed service is shown at Figure 5.

To use as an example of a communication model for a composed service, a real service that can be invoked from our deployment Temperature Control is explained here. Temperature control is a composed service that will evaluate the readings from the environmental temperature (extracted from a node with a Temperature agent deployed) and the body temperature (extracted from the node with the agent that is porting all the data obtained via Bluetooth from a Zephyr Bioharness-marketed belt). The result of the evaluation of temperatures (very high, high, medium, low or very low) is what will be sent to the user. Note that in this case this evaluation of the two separately received temperatures is the data processing stage, for other composed services data processing might represent other actions.

As it was done in the previous case, the followed steps are explained here: the user will request the service (1) as if it was a simple service and therefore there is no difference for the user; it will be invoked the same way, otherwise there would be no real sensor virtualization-. The request will reach the Broker agent, the element of the WSN that has received the service registration from all the other agents (2). As this is a composed service the request will be redirected to the Orchestrator agent, as it is done with all the composed services (3). The Orchestrator agent is incapable of providing a value for that service because it cannot be attended the way that was done before; despite the impression given to the final user, there is no sensor measuring the level of Temperature Control. Nevertheless, the Orchestrator agent is aware that once the values of the environmental and body temperature come to its grasp, they can be processed to have a satisfactory response. So, the Orchestrator agent will ask the only entity of the network that is aware of all the deployed agents -that is, the Broker agent- for the two values that it requires, beginning with the environmental temperature (4).

In this way, the Broker agent will send a request to the Temperature agent deployed in one node (5), asking for the value of the environmental temperature. Once the value is send by the Temperature agent to the Broker agent in a JSON message (6), it will re-route the answer to the Orchestrator agent (7); note that, at this point, the Broker agent neither is aware of the procedure that is taking place at the Orchestrator agent, nor it is expecting another request from the latter agent. However, a single value is not enough to determine the result of the request made by the user (nor it would be sensible to have all this procedure for a single value: it could have been retrieved as a simple service); the body temperature is needed too, so the Orchestrator agent will request it to the Broker agent (8), expecting to be answered as it was done before. And in fact, the request is attended the same way: the Broker agent will ask for the data to the agent that it knows that can provide it, which happens to be the Zephyr agent (9), and this agent will offer it if there is no unforeseen trouble (10). As soon as the Broker agent receives the JSON response with the particular datum, it will send the whole JSON message to the Orchestrator (11), the entity that requested it and is capable of isolating it from all the other content of the JSON message received.

Now that the Orchestrator agent has the two values, it has all the information required to do the evaluation. Once it has taken place, one of the five possible results commented before will be placed into another JSON response message and sent back to the Broker agent (12). Able to distinguish a request from a response, and having as response destination the same node all the times, this Broker agent will send the response to the base station (13), and finally it will be re-routed to the device where the final user started all the process (14).

Additionally, a third kind of service can be obtained in the form of alarms. Alarms are obtained in a way that can be regarded as composed services, with the difference that they will be obtained as information not only when the user asks for it as a regular service (via ESB), but also will spring up for the sportsman/woman performing a sport or some workout when his/her activity reaches hazardous levels (via the Android programmable watch). The storage procedures for alarms are very different depending on whether they are supposed to be received by a human operator having a look at the data retrieved by the services—such as a coach or a sports monitor or by the person wearing the WiMM watch. The Orchestrator Alarms agent will be sending the alarms to the Base station/Sink whenever there is one hazardous value detected, and they will be stored at the PC the Base station/Sink is plugged to by means of a data structure. When the alarm values are requested by a human operator, there is no need to transfer the request to the WSN and add more traffic, for the data will be stored at the PC, and the request will access the stored elements. As for the sportsman/woman, he/she will be receiving the alarmed values almost immediately if coming from body parameters—they will be sent from the node connected via Bluetooth with the Zephyr belt to the mote that is connected with the WiMM watch, and in seconds if they are from environmental temperature—this value will be sent from the Wireless Sensor Network to the mote connected to the Zephyr belt, that is also connected to the WSN by 802.15.4 protocol in order to have him/her monitored.

**Figure 6.**
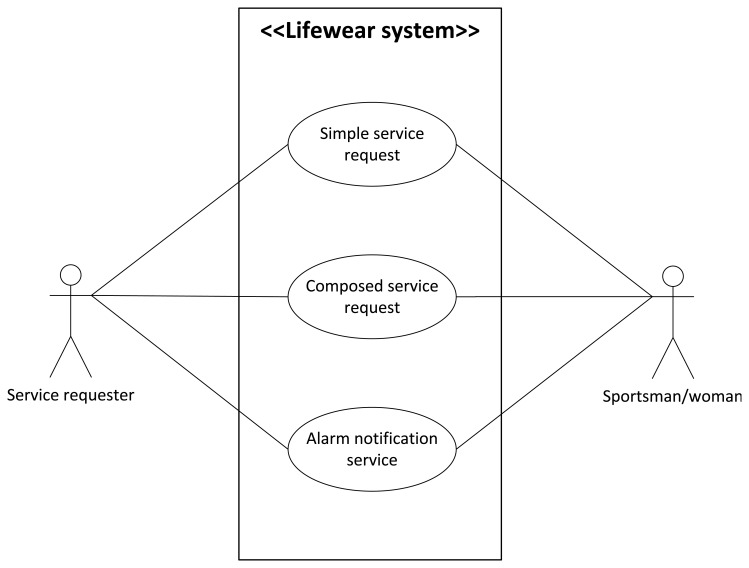
Use cases of the scenario.

In a nutshell, data are provided in second-sizeable periods of time instead of continuously, for it would flood the Wireless Sensor Network with repetitive data—environmental temperature and body temperature are not supposed to change severely in seconds, and if they do so in a hazardous way, an alarm will be triggered. A use case diagram is presented as Figure 6 for better explanation of the services.

The actors involved in this ubiquitous scenario, along with the different use cases presented, are going be extended for them to be completely understood.

#### 3.3.5. System Actors

As it has been established, there are five actors that are part of this system. Those actors are:

- Service requester. They will usually be human beings either requesting simple or composed services from the system. They may also receive alarms, depending on their activities.
- Sportsman/woman. While they can also request for services, they are more likely to receive alarm warnings. Usually, service requesters and sportsmen/women are at least two different users (one requesting services and the other focused on their own activities), but the system could be used for just one person too. The sportsman/woman is being monitored to obtain several body parameters, in a way not dissimilar to a Wireless Body Sensor Network [47], although it is not centred in the human user, as it is just one part of the whole system.

#### 3.3.6. Different Use Cases

The different use cases that were presented in Figure 6 will be exposed in the next sections. Note that since there are several services of very similar nature, they have been gathered under three use cases. Also, only those services registered at the Broker agent will be able to be invoked. If a service has not been registered and it is requested by a user, the request will be dismissed.

- **Simple service request: Temperature request.** This service is used whenever the temperature of the context where the system is located is wanted. As a simple service it can be requested by a human and the Orchestrator agent too, in case it has been required for a composed service.
- **Simple service request: Body temperature request.** This service is used when the body temperature is requested, either by a human user or by the Orchestrator agent, in order to check if there is any alarm going on. This information is collected from the Zephyr belt. When the answer is received, the value and a four digit belt identifier are provided altogether.
- **Simple service request: Heart rate request.** This service differs very little from the previous one; this data will be requested by a human or by the Orchestrator agent, and will be provided by the node receiving the data from the Zephyr belt.
- **Simple service request: Breathing rate request.** Again, this service is provided by the node connected via Bluetooth to the Zephyr device: it requires an external element (the Zephyr belt) and the service requesters will be either the sportsman/woman (rarely, since they will be performing their workout) or the Orchestrator attending a composed service.
- **Composed service request: Injury prevention request.** This, as the next composed service, can only be invoked from a human user. Unlike all the others, this is more likely to be asked by sportsmen/women so it can be checked if they are taking their physical exercises to dangerous levels. The composed service will work like this: when the three required pieces of information are obtained, they are evaluated. If there is at least one of them beyond or below the maximum or minimum thresholds fixed, a High Risk value is sent (and although independently, an alarm will be sent to the ESB and the alarms node). If at least one of the values is within the allowed range, but inside a margin close enough to the upper or lower threshold, a Medium Risk message is sent. Otherwise, a Low Risk message will be sent.
  In order to define the three levels of risk that can be obtained as an answer (see other considerations paragraph), upper and lower thresholds were fixed in our scenario. For body temperature were 38.0° and 36.4° (the Zephyr device was not absolutely accurate when measuring this data and less tight thresholds had to be used), for environmental temperature 34.0° and 12.0° degrees and for heart rate 120 and 50 beatings per minute. Margins were fixed at 5° for environmental temperature, 5 heart beatings for heart rate, and 0.4° for body temperature.
- **Composed service request: Temperature control request.** This service works resembling the previous one, though it requires one less piece of information (heart rate is not taken into account for temperature control purposes). When the two required pieces of information are obtained, they are evaluated. If there is at least one of them beyond the maximum thresholds fixed, a Very High value is sent (and it is very likely that, although independently, an alarm will be sent to the ESB and the alarms node). If at least one of the values is within the allowed range, but inside a margin close enough to the upper threshold, a High message is sent. On the contrary, if at least one of the values is within the allowed range, but inside a margin close enough to the lower threshold, a Low message is sent. Finally, if there is at least one of the values below the minimum thresholds fixed a Very Low value is sent (and again, it is very likely that an alarm will be sent). Otherwise, a Medium message will be sent. The thresholds and margins fixed for injury prevention service were kept for this one.
- **Composed service request: Alarms request.** As the other composed services, this one can only be requested from one final user. This service will notify a user, often different from the sportsman/woman, about a value obtained from the registered nodes that has been beyond the upper or lower threshold values fixed stored inside a JSON message. This received message will contain a three-figured number, where the first digit gives away the nature of the alarm (1 = too low environmental temperature, 2 = too high environmental temperature, 3 = too low heart rate, 4 = too high heart rate, 5 = too low body temperature, 6 = too high body temperature) and the other two give away the value that made the alarm spring up. For example, 235 would be a high environmental temperature alarm (first digit = 2), because temperature is 35° at the room where the sportsman/woman is performing his/her workout. For heart rate the whole figure is added (for example, 338 would be an alarm value claiming that the sportsman/woman's heart is beating at 38 beatings per minute, and 475 would indicate that the sportsman/woman's heart beats at 175 beatings per minute). The value will be visualized when it is requested through the ESB, while an alarm will be sent to the Alarms node by the next service.
- **Alarm notification service.** This is a composed service unique and separated from the others in the fact that it is not requested, but comes up whenever one of the values that are requested (they are the same than those in the previous service) is above or below the thresholds. The alarmed value will be sent to the human user wearing the watch used in our scenario to notify alarms. There is one single agent devoted to provisioning this service (Orchestrator Alarms agent).

**Figure 7.**
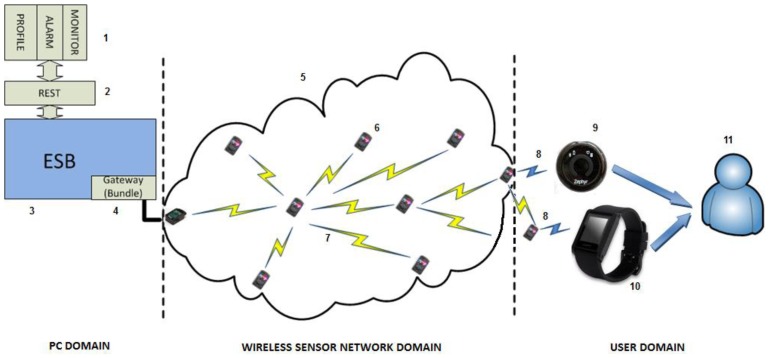
A holistic view of the system.

Figure 7 presents all the elements required for our system.

There are several hardware and software components that must be described in-depth in order to accurately understand how the whole mounted system works.

### 3.4. Hardware Elements of the System

The physical entities present in the system are:

- A Personal Computer with an Ubuntu distribution as the operating system (PC domain). Ubuntu will have an important role in the PC domain, as it is the operating system chosen to install the ESB component that is going to be receiving all the requests from devices belonging to end users. In addition to the ESB (3), the PC will also be mounting the software bundle required to have the base station processing the requests (4) and a REST interface (2) as a gateway between the proper ESB and the elements present in a mobile device.
- A mobile device (or more likely in our particular case, a mobile phone), used to store all the information associated to a particular user, such as the profile (1) height, weight, gender, *etc.* Along with the profile, if an alarm springs up it will be sent from the Wireless Sensor Network (or the node with the Zephyr agent deployed, depending on where it was triggered) to the application storing the profile information, which also happens to monitor the user by requesting the heart rate every two seconds, along with the requests performed to the national Spanish meteorological database (Agencia Estatal de METeorología, AEMET). The software installed in the mobile phone was programmed by SAI Wireless.
- A Wireless Sensor Network (5) behaving as a typical wireless, ubiquitous system. The WSN has nodes scattered in an environment measuring three different temperature values (6). Sun SPOT nodes where used as the hardware of choice for the WSN nodes due to their RAM and ROM capabilities and their low energy consumption, among other features, as it can be observed in Table 1.

Sun SPOT nodes will communicate to each other by using the standard 802.15.4 (7), which is specifying the physical layer and the Medium Access Control (MAC) layers for Personal Area Networks or, in this case, Wireless Sensor Networks.

- At the user domain, the human user (11) will be carrying several devices on. Firstly, two nodes, one with the Zephyr agent that is porting the Bluetooth data from the Zephyr belt, and another one that will receive an alarm notification, should there be any value out of the range fixed for the system by the thresholds (note that while the node with the Zephyr agent deployed can be considered as an endpoint of the Wireless Sensor Network, the other node will not communicate with the WSN at all and will just receive an alarm notification, without sending any piece of information to the WSN. Nevertheless, this node and the former are communicating via Bluetooth data converting boards (8) with the two other user devices: a Zephyr BioHarness™ v3 belt (9) and a WIMM Android programmable watch (10).

According to its data specifications, Zephyr BioHarness™ v3 is a belt capable of measuring different types of human body data [51], and given that it was needed a device that could be worn by a person while doing sport or performing a workout, it suits fine for our purposes. This belt is the device collecting the body-related parameters used for our system (body temperature, heart rate, breathing rate) but since the data are transmitted via Bluetooth and Sun SPOT nodes do not support it natively, their hardware had to be augmented by using Bluetooth data converting electronic boards. An electronic board model, suitable enough due to its size and its capabilities, is marketed by Sparkfun Electronics—model Bluegiga WT-32, so two Bluegiga boards were purchased [52]. One board was attached to the node that was charged with the task of converting the Bluetooth required parameters into data that could be transferred throughout the Wireless Sensor Network; the other to the node that would communicate with the WIMM programmable watch.

Finally, another device was required to notify the user of any alarm that would come up regarding his/her physical conditions (too high heart rate, too low body temperature, *etc.*). To accomplish this task, another device was purchased: a programmable Android watch from a vendor named WIMM (the watch has been named WIMM One) [53]. What was interesting for our deployment is that this watch could be programmed to have events notified: if, for example, there was an alarm regarding a too high heart rate, the watch could be programmed to display it at its LCD screen, along with a beeping sound to warn the final user, as it was finally done.

### 3.5. Software Elements of the System

There were as many software elements present on the system as hardware ones. Most of them were agents with purposes related strictly to the Wireless Sensor Network; others were out of the Wireless Sensor Network but inside the system nevertheless.

**Figure 8.**
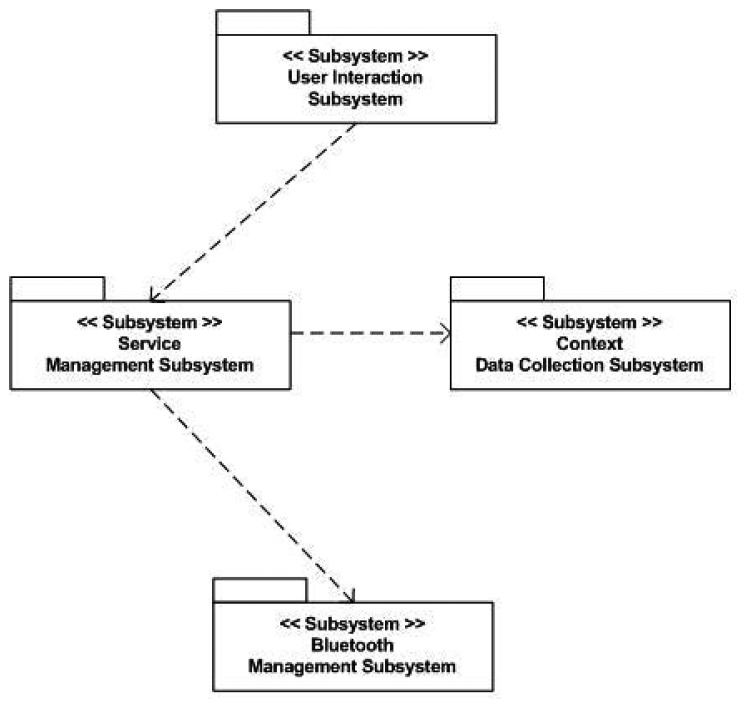
Subsystems diagram of our system.

From a software perspective, our architecture can be separated into four different subsystems, each one with a different concern, as depicted in Figure 8. User Interaction subsystem will be focused on all the duties related to the successful retrieval of requests from the user. Service Management subsystem will be bent on taking the necessary actions to obtain the requested information from the Wireless Sensor Network. Context Data Collection subsystem will collect the information that is related with the environment where the system is deployed. Finally, the Bluetooth Management subsystem will be taking care of all the issues related with data collection from the Zephyr belt, and alarm delivery on the node that is connected via Bluetooth to the Android watch. The subsystems are relating each other in a particular way: the User Interaction subsystem will ask for the information to the Service management subsystem, which will have gathered it from requests done to Context data Collection and Bluetooth Management subsystems.

#### 3.5.1. User Interaction Subsystem

**Figure 9.**
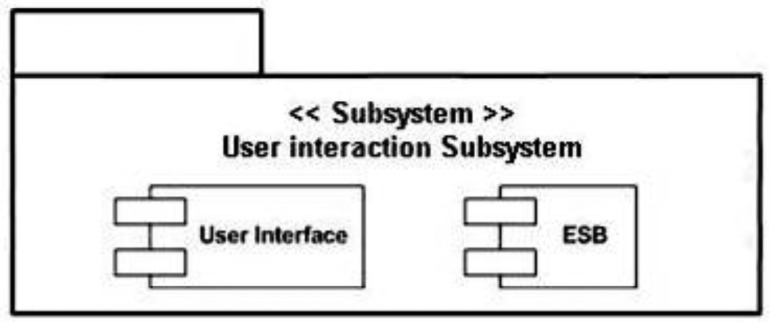
User Interaction subsystem and its inner components.

It can be observed in Figure 9 that there are two components present in this subsystem, the ESB and the User Interface.

**Figure 10.**
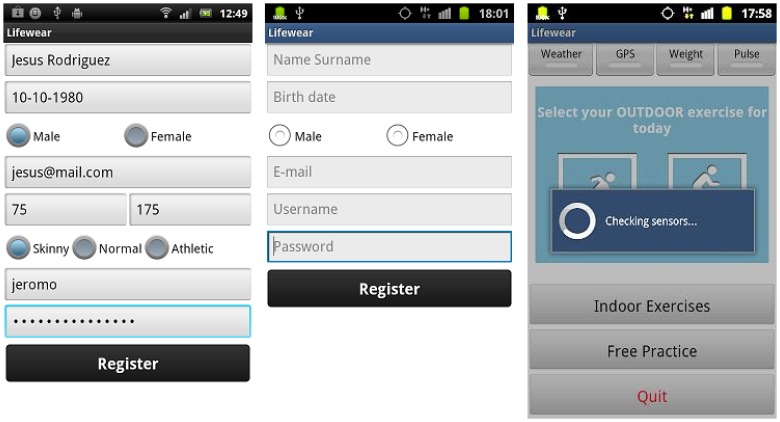
Graphic User Interface of the Lifewear mobile application. Profile registration and body sensor checking procedures.

ESB (numbered as 3 in Figure 7) is an acronym for Enterprise Service Bus. It is a software architecture model under the principles of Service Oriented Architecture that allows the integration of different technologies used by separated service invocators. In a way, it plays a role resembling that of the middleware: it will interpret the requests that it receives and, as long as they are in a format understandable by the ESB, service requesters will not have to worry about delivering their petition in a particular format, since they will be interpreted by the ESB. Once the ESB receives the request, it will resend it to one of the interfaces it has to communicate with an internal system, either to a PC or to a mobile Graphic User Interface tailored for the system, as shown in Figure 10.

In our system, the ESB will be useful to homogenize the nature of the requests done by the final user: it has been tested with petitions originating from tablets, mobile phones and the PC where it is installed.

The User interface, on the other hand, refers to the way the user requests access the ESB. It is done by two ways: On the one hand, the services can be accessed via URL, where all the information related to the required IP address, the port number and the service that is going to be consulted is included. For example, if the Injury prevention service is invoked, the URL would be: 192.168.0.199:8181/cxf/crm/ lifewear/injury. This URL was using a private IP direction because it was for testing purposes; when the Lifewear scenario was deployed, or external tests had to be undertaken, a public IP address was provided.

On the other hand, a Spanish SME called SAI Wireless [54] devised an interface to access several services as part of their own developments in the Lifewear project. The interface was thought to be offered as support for a sports routine, and would advise the user to perform warming ups, different workouts, *etc.* Before any workout may get started, a user profile is created and sensors present in a room are checked.

**Figure 11.**
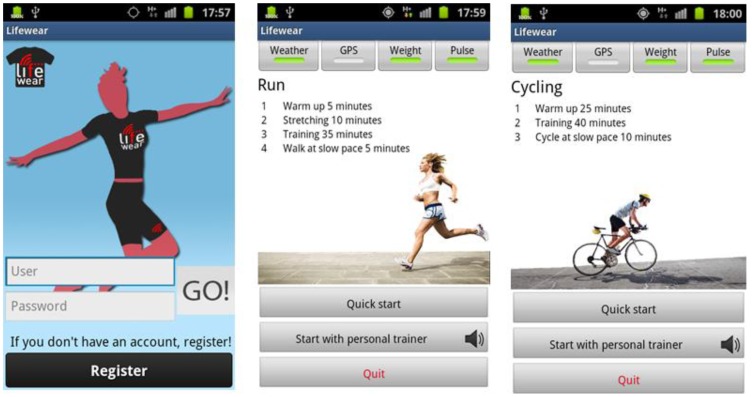
Graphic User Interface of the Lifewear mobile application. Login and training screenshots.

The user interface that has been developed by SAI Wireless will offer an interface regarding all the services that can be obtained from an application perspective. These services involve a training routine that will vary depending on the choices of the final user: for example, the layouts for the interface regarding jogging and cycling were designed, and a login service was created before these services can be accessed by a user profile as the one shown in Figure 11.

**Figure 12.**
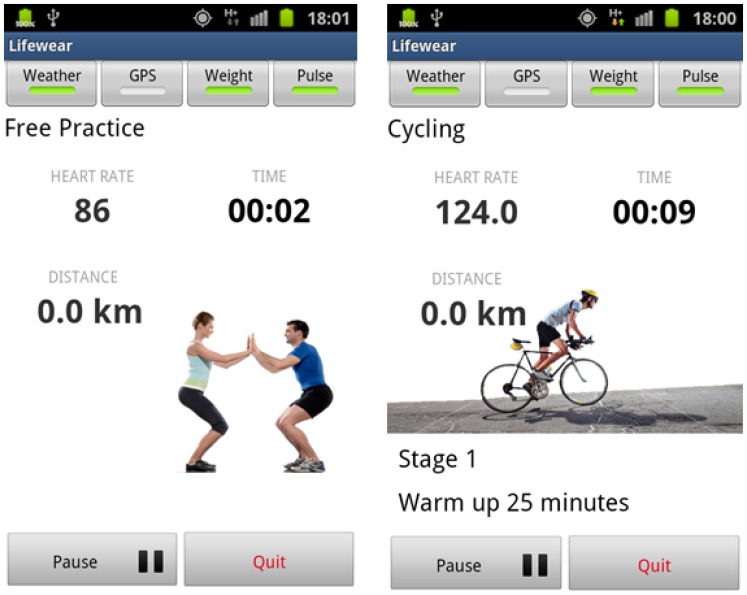
Graphic User Interface of the Lifewear mobile application. Real time data.

In addition to that, SAI Wireless application will also receive output data from the services belonging to the system deployed in the gymnasium, such as heart rate or risk of suffering an injury, along with real time data, as presented in Figure 12.

#### 3.5.2. Service Management Subsystem

**Figure 13.**
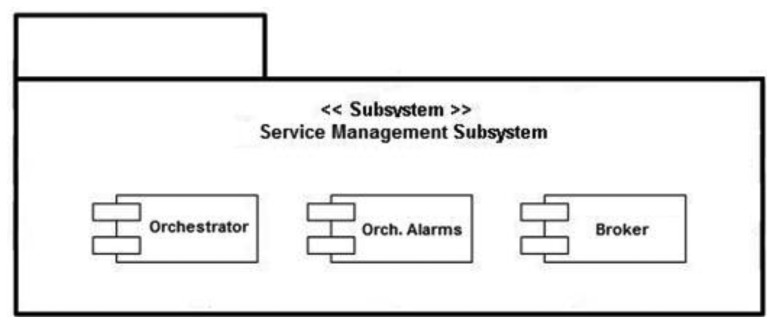
Service Management subsystem and its inner components.

This subsystem has three components, as displayed in Figure 13.

**Figure 14.**
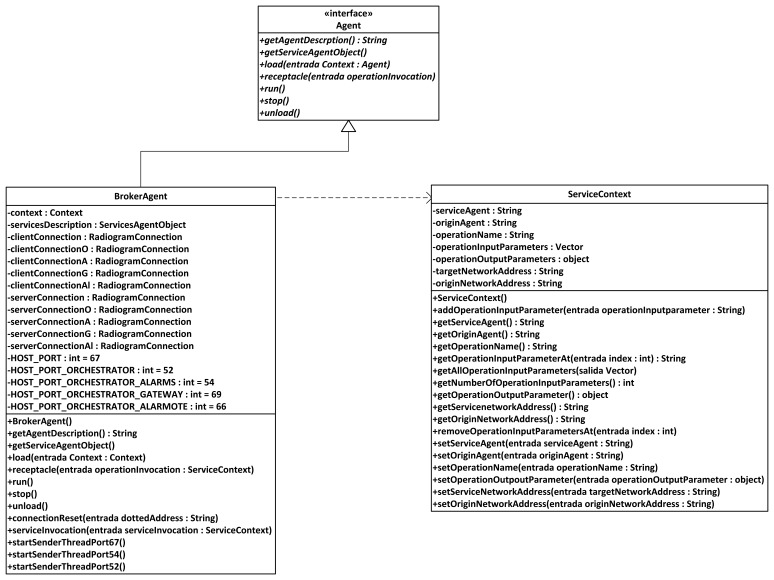
Class diagram of the Broker.

The functionalities of these components are what can be expected from them: the Orchestrator agent is the key component in composed services processing, requesting the Broker agent for the simple services that will be asked for, and the Broker agent is critical for the whole semantic middleware architecture and the Wireless Sensor Network once it is deployed, since it has registered which services have been announced and all the requests and responses of the Wireless Sensor network must go through it, at one point or another. The Orchestrator Alarms agent is the one that will be requesting all the needed data to check whether a value is “alarmed” (that is, above or below the values fixed for the upper and lower thresholds). The Broker agent most relevant classes are depicted in Figure 14.

**Figure 15.**
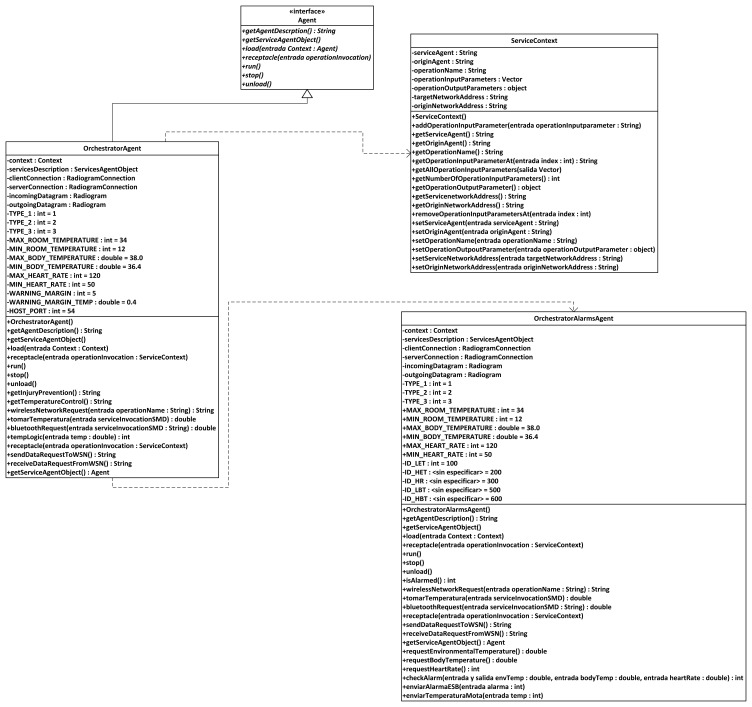
Interrelating class diagram of the Orchestrator and Orchestrator alarms agents.

Figure 15 depicts how the other two components are interrelating in a similar fashion.

#### 3.5.3. Context Data Collection subsystem

**Figure 16.**
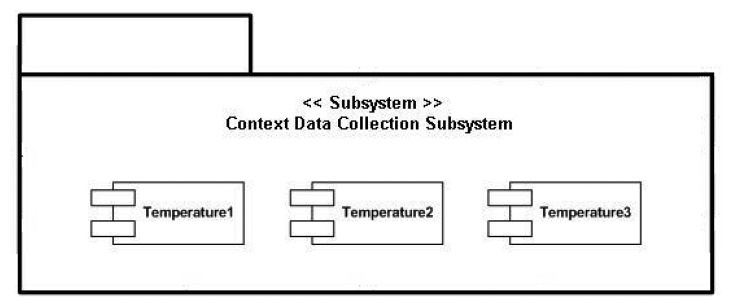
Context Data Collection subsystem and its inner components.

This subsystem is responsible for measuring and providing the environmental information that is proper of the environment the WSN is deployed in. It has three similarly working components that can be seen in Figure 16.

**Figure 17.**
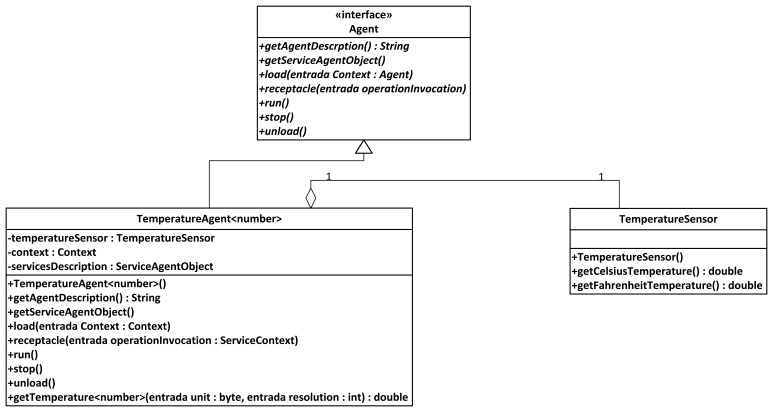
Class diagram of the temperature agents.

There are three temperature agents that will be deployed in three nodes, since the temperature in three different places of the environment was measured. Plus, each of the equally functional agents was under different conditions, so it is a good way to test what sort of disturbance is the worst for the network. Although there are three different Temperature agents, they essentially work the same way and are using the same classes (see Figure 17 for further details).

#### 3.5.4. Bluetooth Management Subsystem

**Figure 18.**
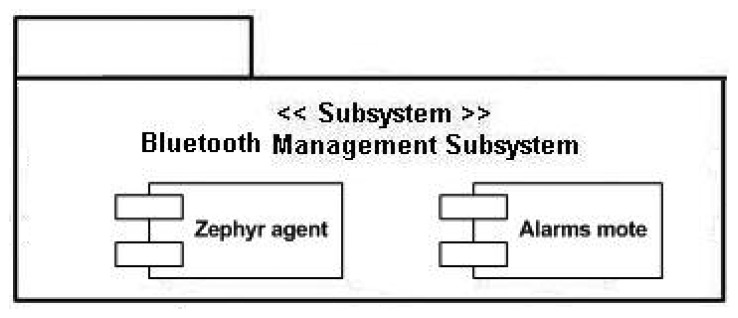
Bluetooth Management subsystem and its inner components.

This last subsystem has two more components, as it can be seen in Figure 18.

**Figure 19.**
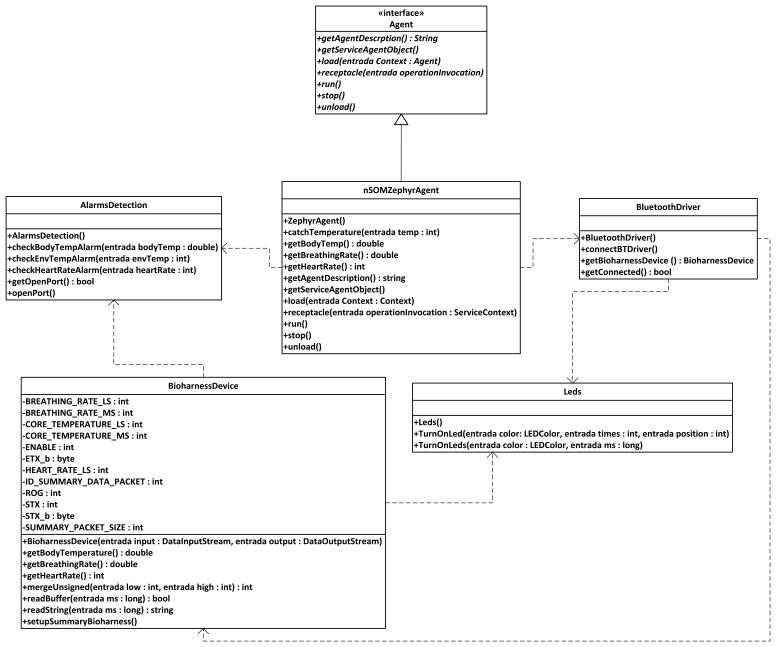
Interrelating class diagrams of Zephyr and alarm agents.

The two components are going to deal with their already known functionalities: the Zephyr agent will adapt the data obtained from the Zephyr belt via Bluetooth connection to a format that can be understood by the other nodes, and the Alarms node will be taking the alarm information to the Android programmable watch via Bluetooth communication as well. A class diagram referred to this subsystem is offered at Figure 19 for a more accurate knowledge of the system.

### 3.6. Communication on the Application Layer of the System

The communication is going to be tackled by using JSON (JavaScript Object Notation) messages. Apart from the particular format required by JSON, an additional one has been establish in order to have a standardization of the communication that covers the field the WSN will be operating, and in this way the particular data can be recovered in an easier way.

The request message that will be sent from the base station throughout the Wireless Sensor Network will be formatted as follows:
{“transport”: “j2me.radiogram”,“envelope”: “JSON-2.0”,“target”: “<IP or MAC address>/<name of the destination agent> ”,“origin”: “<IP or MAC address>/<name of the source agent>”,{“operation”: “<operation name>”,“parameters”: [< parameters or void, if there are none>]}}

The JSON response message will have a very similar layout, being the only change that a new field named “result” will be placed under the parameters of the requested service. When an alarm is requested, the answer brought to the final user via ESB will be a JSON message that has a parameter the value that has triggered the alarm.

**Figure 20.**
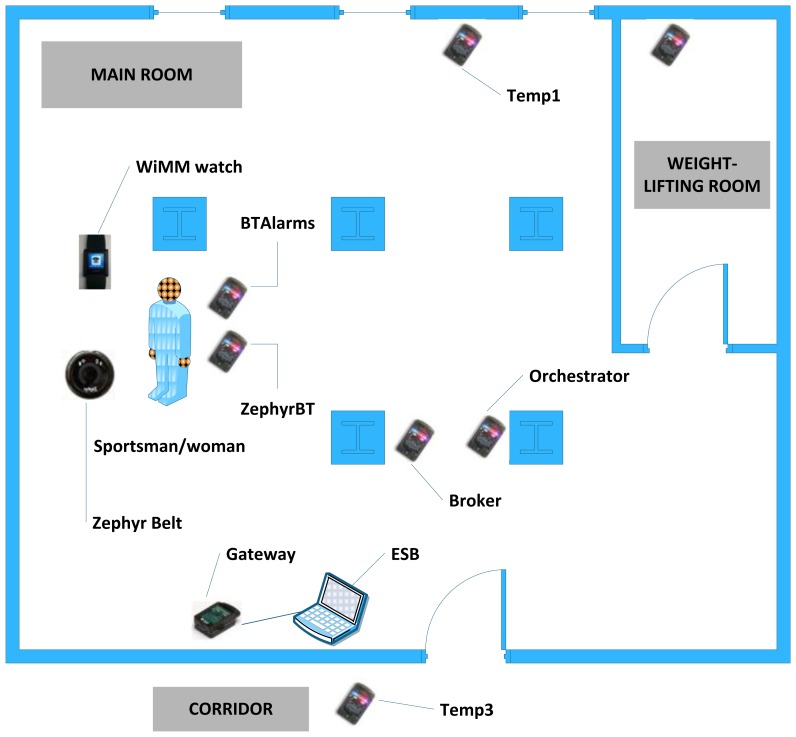
Gymnasium deployment of the system.

All these hardware and software elements were deployed in a gymnasium located as part of the premises of UPM in a fashion that is displayed in Figure 20. The hardware elements were deployed as a way to balance coverage area and reliability: while the temperature nodes were positioned where they could be most useful, that is, scattered at the walls of the different premises the main room of the gymnasium, the weight-lifting room and at the corridor outside, the nodes responsible for purely software and communication tasks where stuck to two pillars at the center of the main room, so as to be able to communicate with all the different elements of the room with no radio-related issues. Other configurations that involved having the PC where the ESB was installed at the weight-lifting room, or having all the temperature motes at only the furthest wall instead of each of the nodes in a different one, were discarded for being either impractical or providing redundant information.

## 4. Results and Discussion

Once the scenario was mounted and the agents uploaded to the nodes, several performance tests were carried out. Results, according to which services and features were tested, have been included.

### 4.1. Battery Consumption of the Different Nodes of the System

A test meant to know for how long a node would be turned on until its energy was completely depleted was carried out, without making any request and letting the only energy that was consumed be the one required by the most basic performance of the Wireless Sensor Network. Although all the nodes were initially fully charged, due to the different amounts of energy required by their different roles, the lifespans involved were usually dissimilar, as it has been shown at Table 2.

The gathered data reflect the conditions nodes had to work under. Nodes with the temperature agents deployed where the most durable ones, since they had either very low (Temperature 1 and Temperature 3) or relatively low (Temperature 2) traffic load. Temperature 2 node was requested environmental temperature every short period of time, but these requests were from the orchestrator node (the one with the Orchestrator and Orchestrator Alarms agents uploaded; the latter was the one making the requests), and once it turned off, no more requests reached the node with the Temperature 2 agent deployed, thus resulting in a longer than expected lifetime. The node acting as the broker (for it had the Broker agent uploaded) would run out of battery sooner, again due to the requests coming from the orchestrator node not only about environmental temperature, but also about body temperature and heart rate. Nevertheless, the orchestrator node was not the first one to run out of energy; due to the technology used by the Zephyr Bioharness belt, regular Bluetooth had to be employed for communications, instead of the Low Energy Bluetooth standard. This, added to the usual amount of energy depletion due to Wireless Sensor Network requests and responses, resulted in having the Bluetooth-connected alarms as the ones with the lowest lifespan. Particularly, the node connected to the Zephyr belt was the least durable one, for it was often requested about parameters that could only be obtained by transmissions taking place through it. The Alarms node was not required to act all the time, so a slightly better lifespan was obtained in this case.

Judging from the results already displayed, the impact of service orchestration is notorious in the node that is in charge of service orchestration and alarm-related duties (which are using several pieces of data and can be regarded as composed services), since its lifespan is 69.3% of Temperature 1's and 2 hours below the other node performing non-measurement tasks (the one with the Broker agent uploaded). This is due to the many data radio transmissions and receptions needed for composed services, that are usually the most energy-costly operations for a node in a Wireless Sensor Network.

### 4.2. Setup of the Wireless Sensor Network

Here, it was measured how long it would take for the whole Wireless Sensor Network to be set up and have it in full working conditions. In order to perform the test, all the required nodes and the Zephyr belt were turned off to begin with, and they were progressively turned on. Banal as it may seem, it is a delicate procedure because the nodes cannot be reset or turned on at the same time, or without any order: obviously, if the Broker is the last node to be reset or turned on, the Wireless Sensor Network will not work at all because not a single service will be registered and all the requests will be systematically dropped. To have a successful setup, the node with the Broker agent deployed must be the first one to be turned on or reset; after it, it is advisable to turn on the Zephyr belt first and the node with the Zephyr agent uploaded right after that. Then the node with the Orchestrator and the Orchestrator Alarms agents must be reset, and finally the nodes with the temperature agents.

There are some other considerations that have to be made: the node with the Orchestrator Alarms agent deployed (which will usually have the Orchestrator agent deployed as well) is the most verbose of the network by far. This is so because Orchestrator Alarms agent does not wait to be invoked by a user, but every five seconds (or in case of heart rate, one second) is asking the WSN for the data that it requires so as to make sure of the existence—or inexistence of a value out of the bounds marked by the thresholds. Another challenging node is the one with the Zephyr Bluetooth agent deployed; while this node does not add much traffic to the WSN, if the agent fails to be registered a lengthy series of actions will have to be done to give it a try again (turning off the Zephyr belt, waiting 10 seconds, turning on the belt again and finally resetting the node) due to the behavior of Bluetooth connections, thus adding a considerable delay in the network setup.

In order to have reliable results each of the made tests was done by taking twenty five measures. The most significant results are depicted in Table 3.

**Figure 21.**
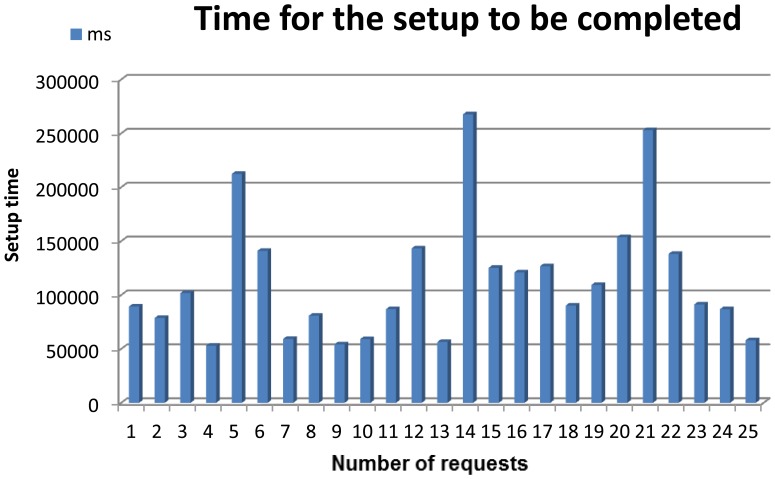
Time used in setting up the WSN (25 attempts).

The small but significant difference between the average and the median values is indicating that there is certain heterogeneity with the obtained results, and actually, there are only two readings that are around 90 seconds; the disparity among them is widespread. This is due to the fact that several attempts were conditioned by having had an agent failing to register its first time, and therefore the required time at that measure soared, especially if that issue had happened with the Zephyr agent, or if the Orchestrator Alarms agent was overwhelming the Wireless Sensor Network. The heterogeneity of the obtained values can be observed in Figure 21.

### 4.3. Simple Services Analysis: Temperature Services

Once the Wireless Sensor Network had been setup, the provided services performance could be tested. There were three different temperature readings that could be obtained from the environment, since there were three agents deployed onto three different nodes, so the obtained results were not be the same for the three of them, because the nodes with the different temperature agents had been tested under different conditions:

- The node with the temperature agent named Temperature1 was the furthest away from the Broker; it was wanted to know how this would affect the communications. In order to have 25 successfully answered requests, a total of 27 had to be made. This failure rate, as low as it may be (2 out of 27 attempts), is the highest of the three temperature agents tested.
- The node with the temperature agent named Temperature2 deployed was under different conditions than the former: it was relatively near the node with the Broker agent, but its services were requested by the Orchestrator Alarms agent for the environmental temperature every little time, so it was under an acceptable but constant stress. Apparently, though, it did not affect the performance of the agent because only one request failed to be attended (1 out of 26 attempts). The obtained time values were slightly better than with Temperature1, further away but with no other requests that the ones made by the user.
- The node with the Temperature3 agent deployed on its hardware was given a third different environment: it was at a closer distance than the node with Temperature1 agent deployed, but further away from the Broker agent than Temperature2 node. However, it did not attend any other request than the ones that were made by the human operator during this testing session. Therefore, it should come not as a surprise that the 25 requests were attended without any failure (failure rate: 0/25 attempts).

The most significant results have been gathered in Table 4 in order to have a safe ground for comparison between the services that are being provided.

**Figure 22.**
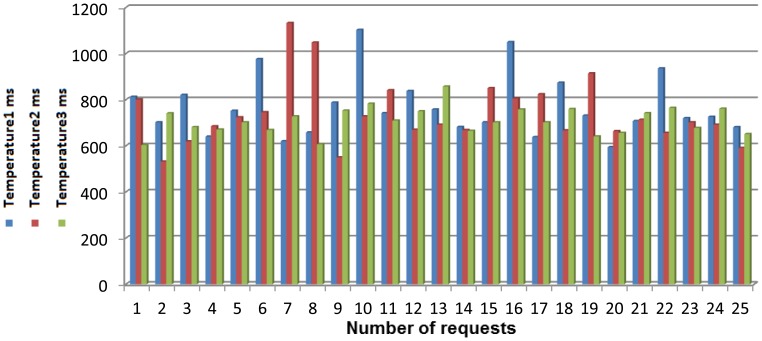
Time used to answer temperature requests.

There are some remarkable features that must be highlighted from the collected information. As far as Temperature1 readings are concerned, the disparity between the average value and the median, albeit small, is significant. This is due to the fact that some of the requests took a longer than usual time to be attended, thus adding some irregularity to the general results. Temperature 2, although having a constant influx of requests from the Orchestrator Alarms agent has a lower difference between the average and the median values when compared to Temperature1. This is pointing out at the fact that the data are evener and are confined in a narrower values range. Finally, average and median values from Temperature 3 are remarkable when compared to the ones obtained from Temperature1 and Temperature 2 agents: not only these values are slightly lower, but also the average and the median values have reduced their difference extraordinarily; clearly, having a node without issues related with the power of the radio signal, and without the disturbance of having to attend requests from two sides (a human user and an inner agent) pays off in performance terms. A better understanding at the obtained results can be gathered from Figure 22.

The results from Figure 22 highlight in a more visual way what was mentioned before. Temperature 1 requests will be resolved in a higher amount of time, and will also be the ones that most usually will require periods of time way over the average. Temperature 2 is offering the most uneven results, although it offers an overall more stable performance than Temperature 1. Temperature 3 is the service showing the less varying results; all in all, it is the most reliable service, due to the established conditions to have it measured.

### 4.4. Simple Services: Analysis of Body Temperature Service

This service has a strong difference with the others, despite being a simple service like them: it is not depending on a “local” sensor to retrieve the data, but it has them sent from the Zephyr belt via Bluetooth transmission. Since the Zephyr device will transmit data every second, a performance less appealing than the ones of the temperature agents should be expected. But actually, although results are worse when compared to the other nodes, they do not pale in comparison. In order to have 25 requests successfully answered, 27 had to be taken (error rate: 2/27 attempts), just like it happened with the furthest away node, the one with the Temperature1 agent deployed. The most significant obtained results are presented in Table 5.

**Figure 23.**
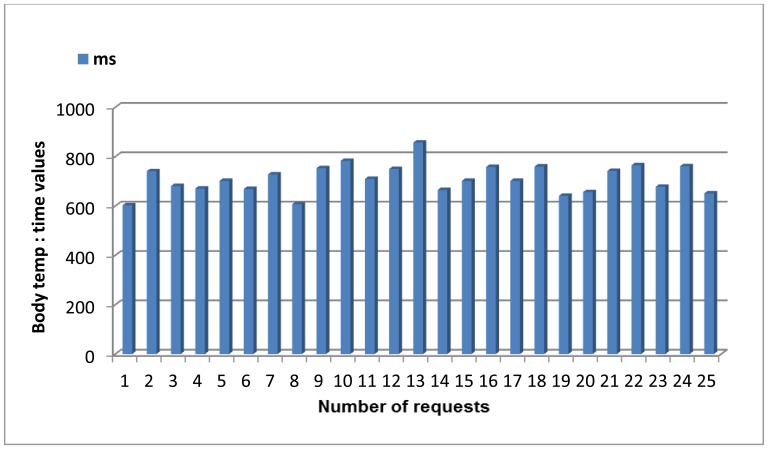
Time used in completing body temperature request (25 attempts).

Considering that the average (775,296 milliseconds) and the median times (755 milliseconds) are higher than in the other cases, and the difference between the average and the median value increases—thus, the results are less homogeneous than before the lower expectations about this agent are confirmed. However, it has to be given credit for not lowering the performance significantly, especially taking into account that the agent is dependent on an external device to harvest the data from the environment. A graph with the carried out tests is in Figure 23.

### 4.5. Composed Services Analysis: Injury Prevention Service

To offer a wider view of the general performance of the system, the same analysis procedure has been applied on the composed services of the system.

**Figure 24.**
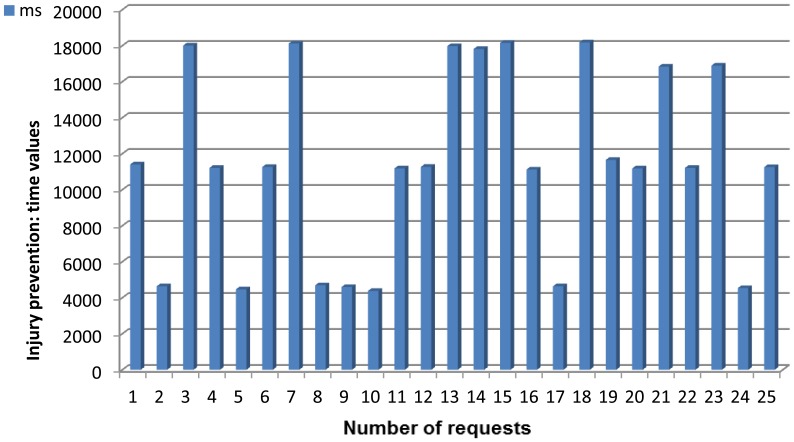
Time used in completing injury prevention service request (25 attempts).

Injury prevention service fares the worst in terms of performance (see Figure 24) due to two reasons: firstly, it requires the retrieval of three pieces of information to be fully and successfully delivered; secondly, the service is requested under not so favourable conditions, with another element of the Wireless Sensor Network (the Orchestrator Alarms agent deployed altogether with the Orchestrator node) sending requests for information in a fast pace. Its reliability is affected as well: out of 28 requests done, three failed (error rate: 3/28 attempts); it is the worst result of all the tested services. Consequently, the average time required to serve this service is way longer than what it was with simple services (even when all the requests for the simple services are summed up), as it can be learnt from Table 6.

There are two facts that must be considered about these measures: firstly, the results obtained are much worse than in simple services. Plus, a pattern can be learnt from here: data collected are showing periods of time around 4.5, 11 and 18 seconds. This is due to the fact that the Broker and the Orchestrator agent are competing against the Orchestrator Alarms agent to get the data, and in this competence they can be completely successful (pieces of information for the injury prevention service will be obtained before the data for the alarm checking procedure, thus taking for the service around 4.5 seconds to be served), mildly successful (the Broker and the Orchestrator agent must wait for the Orchestrator agent to fish one data request, thus serving the injury prevention service in around 11 seconds), or not that successful (the Broker and the Orchestrator agents have to wait even more time).

### 4.6. Composed Services Analysis: Analysis of Temperature Control Service

This composed Service requires less information pieces to be composed, and therefore its performance is better than the former case. Its reliability is slightly better too: out of 27 requests, only 2 failed to provide the service (error rate: 2/27 attempts). The most relevant results are displayed in Table 7.

**Figure 25.**
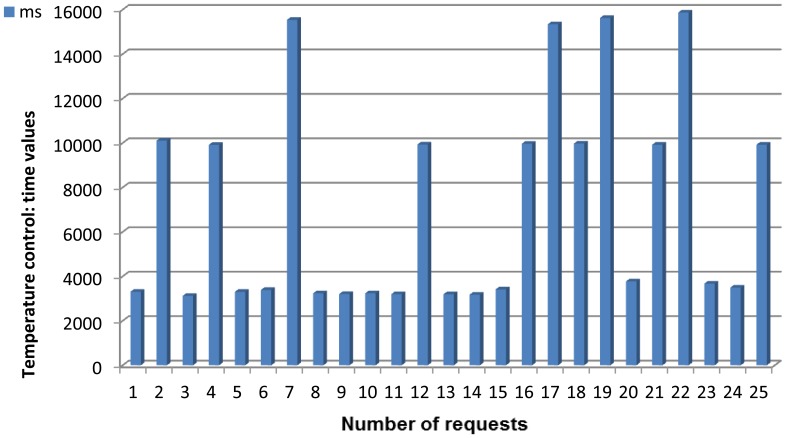
Time used in completing temperature control service request (25 attempts).

Since now only two simple services are required to compose the third one, the times around the services are delivered are 3, 10 and 15 seconds. All these facts can be seen in Figure 25.

### 4.7. Reset Time Analysis

One more test that is going to be offered is regarding the time required for an agent to get successfully registered at the Broker agent. As the reliability of Wireless Sensor Networks is one of their most important features to be considered, it seems interesting to know how long would take an agent to register itself again if the node it is deployed on has gone down for any reason (data floods, energy depletion, *etc.*). In this way, it is considered that the reset procedure is completed when the uploaded agent of a node is re-registered. The most significant results obtained are presented in Table 8.

**Figure 26.**
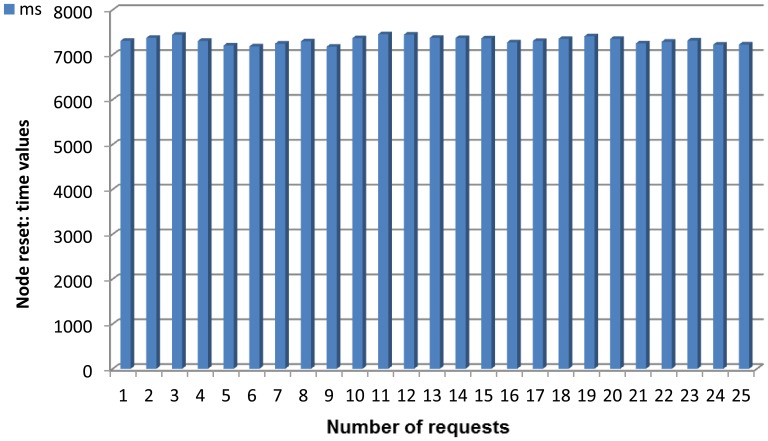
Time used in completing a node reset (25 attempts).

The data obtained are very homogeneous (in fact, the average and the median values are 7316.298 milliseconds and 7,309 milliseconds, proportionally, the least differentiated of all the tests done). It can almost be concluded that the agent is working by using the exact same amount of time. The data represented in the chart above these Lines has been poured into the Figure 26.

### 4.8. Connection time Between Zephyr Bluetooth Agent and Bioharness Zephyr Module

The required time for the node equipped with the Zephyr Bluetooth agent to establish a connection with the Bioharness Zephyr module (that is, the part involving the Zephyr belt) has been tested too. In order to make the connection possible, the Zephyr belt has to be turned on before the node with the deployed agent is done so, for the Zephyr Bluetooth agent must recognize Bluetooth data in order to successfully establish the connection. A chart where the most significant measures are presented is in Table 9.

**Figure 27.**
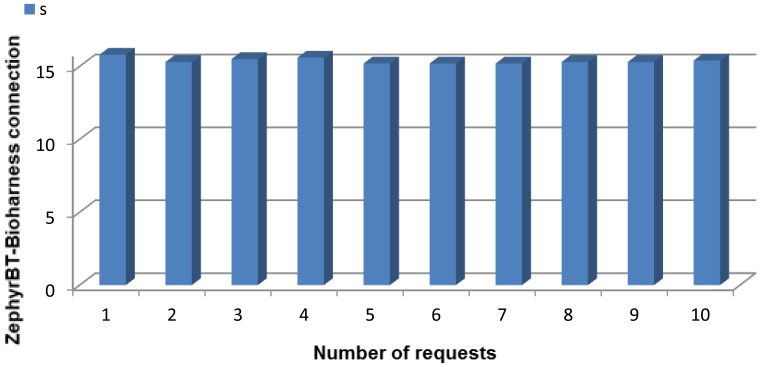
Time used in establishing a ZephyrBT-Bioharness connection (10 attempts).

Alternatively, a graph has been created by using the test results throughout the 25 times connection time was tested; it can be seen at Figure 27.

### 4.9. Alarm Reception on WiMM Watch

Finally, after an alarm has sprung up because of the data measured either as context information (environmental temperature) or from the sportsman/woman (heart rate or body temperature), the latter must be notified about it. In order to accomplish this task, a Bluetooth-enabled Android watch was programmed to receive both the alarm notification (a beeping sound that would differ depending on the kind of alarm sent to the watch) and the measured value that triggered the alarm (deployed in the watch screen as well). This alarm will be transmitted from the Zephyr belt to the Zephyr Bluetooth agent node, and via 802.15.4, from the node with the Zephyr Bluetooth agent deployed to the one with the alarms Bluetooth agent and from here to the WiMM watch via Bluetooth. The most significant results are shown in Table 10; note that the depicted figures deal with the total amount of consumed time from the instant when the Zephyr belt reads the alarming value to the instant when the WiMM watch displays it on its screen.

**Figure 28.**
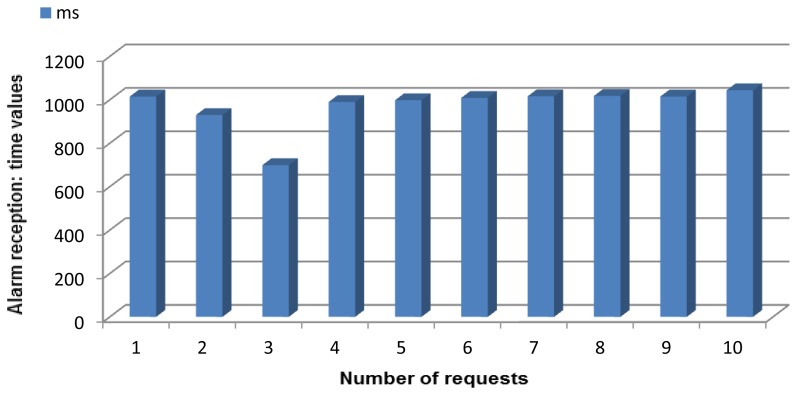
Time used in transferring an alarm from the node to the belt (10 attempts).

A graph has been created again so as to have a more visual impression of the gathered information about this test; it is Figure 28.

If a glimpse is taken on the data, it can be observed that once the alarm has been triggered it will usually take around one second to be transferred to the Android programmable watch. For a sportsman/woman we consider this value to be acceptable as the required time to notify of the alarm, since the thresholds the system is working under are low enough to guarantee that if the person performing his/her workout is having hazardous activity levels, they will be notified way before serious consequences appear, like muscular injuries or fainting.

## 5. Conclusions and Outlook

It has been proved throughout this paper that applications based on the Internet of Things, and more specifically, running on semantic middleware architectures, are feasible not only as a theoretical model but also as a practical implementation, such as the system deployed in the UPM's gymnasium facilities. Our system has provided a final human user with a set of services with useful functionalities about context information and body parameters for an indoor scenario where exercising routines or sports can be performed. From a purely technical and research-oriented point of view, very different technologies (Standard 802.15.4, standard Bluetooth, Sun SPOT nodes and their equipped sensors, Java 2 Micro Edition, *etc.*) have been integrated in the same system, so seamlessly that the different components are almost unnoticed by a final user. What is more, regardless of the heterogeneity of the technologies, our semantic middleware proposal manages to operate satisfactory the system, when requests or alarms have to be tackled. From a human point of view, it is expected that by using this and other systems resembling ours, sportsmen/women performance can be better evaluated, either if they are used in an elite environment or for the elderly when they feel like performing a sport. Finally, from a commercial perspective, it has been proven that building applications and systems related with the Internet of Things can be exploitable and profitable, as it can be inferred from the collection of companies that have taken part in the Lifewear research project.

As for the data obtained from the test benches executed, there are some other conclusions that can be inferred. For example, regarding simple services it is interesting to note that distance (and therefore, the strength of the signal that is communicating with the nodes) is more of an issue than a moderate load of traffic when requests coming from a user have to be responded. The requests will fail somewhat oftener and the ones that are delivered will be slightly slower and less reliable if one node is too far away. Obviously, the lesser traffic a node has to deal with and the nearer it is (but not so near to have interference phenomena), the better its performance will be. It should also be pointed out that when an external factor is put into use in a Wireless Sensor Network (in our case, a Bluetooth-enabled belt), it is quite probable that it will lower the performance of the WSN element it attaches to, because it will make it dependent on the pace of the external device in its data deliveries. Nevertheless, if it has to be done, Sun SPOT nodes have proven not to crash easily.

It has to be born in mind that all the tests that are shown here were done with the nodes fully charged, so battery levels should not be an issue when comparing performances among services. For future developments, it would be extremely interesting to spread the usage of this system to several people instead of one sportsman/woman. Since an identifier has been provided for a particular device, it is a feasible possibility to implement a new system involving several persons.

There are several conclusions regarding how composed services are offered in our system that should be taken into account as well: composed services take some punishment from the almost constant activity of the Orchestrator Alarms agent: since they require a lot of messages until finally completing their tasks (especially if many pieces of simple information are required), they are prone to suffer from delays at any of the links needed to have a fully functional chain of requests and responses, both inside and outside the domain of the Wireless Sensor Network. Nevertheless, the performance of a composed service, albeit worse than that of a simple service, is at least fairly predictable: depending on how fast the requests for simple data were attended the result will be obtained around very specific values.

Although our semantic middleware architecture has proved to have a high level of maturity through the Lifewear project, and can be regarded as noteworthy in the fields of ubiquitous computing and semantic, pervasive middleware by its own right, there are still several improvements that could be interesting to tackle in future versions, judging from the results that have been obtained in the real scenario: if this architecture is going to be used in a wider area, where there are many rooms and many places, a location system could be useful. Additionally, a GUI-based software management application to better handle code from/to the nodes would be another improvement to think of.

## Figures and Tables

**Table 1.** Sun SPOT node technical features as of its latest release [48,49].

Central Processing Unit		Interfaces	
Microcontroller	AT91SAM9G20	Types	Digital (GPIO), analogical (ADC), serial (I²C, I²S, SPI0, SPI1, USART)
Memory	1Mbyte RAM, 8Mbytes Flash ROM	Sensors and actuators	Light, Temperature, Accel., 8 LEDs, 2 switches.
Operating System	None (Squawk virtual machine)	Gateway Infrastructure	Mini USB 2.0
Radiofrequency		Others	
Transceiver	CC2420	Battery	3.7 V, 770 mAhr Li-Ion rechargeable battery
Data speed	250 Kbps	Power usage (deep sleep/active)	33 uA/104 mA
Range	100 meters (outdoors, antenna-equipped)	Price [50]	314.93 € per kit (two nodes and a base station)

**Table 2.** Time for a node to deplete its battery.

**lifetime**	**hours**
**Temperature 1**	**20,2**
**Temperature 3**	**20**
**Temperature 2**	**19**
**Broker**	**16**
**Orchestrator**	**14**
**Alarms**	**8,2**
**Zephyr**	**7**

**Table 3.** Most Significant Measures Obtained for WSN Setup.

**Request**	**Time for the setup to be completed (ms)**
**Average**	92337.7266
**Median**	91257

**Table 4.** Most significant measures obtained for Temperature services.

**Request**	**Parameter**	**Value of temperature parameters (ms)**
**Temperature 1**	**Average**	751.207237

	**Median**	731
	
**Temperature 2**	**Average**	718.515800

	**Median**	702
	
**Temperature 3**	**Average**	704.760545

	**Median**	702

**Table 5.** Most significant measures obtained for body temperature service.

**Request**	**Time for the body temperature request to be responded (ms)**
**Average**	775.296622
**Median**	755

**Table 6.** Most significant measures obtained for injury prevention service.

**Request**	**Time for the injury prevention request to be responded (ms)**
**Average**	8702.68164
**Median**	11257

**Table 7.** Most significant measures obtained for temperature control service.

**Request**	**Time for the temperature control request to be responded (ms)**
**Average**	4839.65438
**Median**	3672

**Table 8.** Most significant measures obtained for an agent to get re-registered.

**Request**	**Time for a node reset to be completed (ms)**
**Average**	7316.298
**Median**	7309

**Table 9.** Most significant measures obtained to establish a ZephyrBT node-Bioharness belt.

**Request**	**Time for a ZephyrBT-Bioharness connection to be completed (s)**
**Average**	15.38
**Median**	15.3

**Table 10.** Measures obtained to establish a ZephyrBT node - Bioharness belt.

**Request**	**Time for an alarm to be delivered (ms)**
**Average**	973.4
**Median**	1012
